# Positive selection in admixed populations from Ethiopia

**DOI:** 10.1186/s12863-020-00908-5

**Published:** 2020-10-22

**Authors:** Sandra Walsh, Luca Pagani, Yali Xue, Hafid Laayouni, Chris Tyler-Smith, Jaume Bertranpetit

**Affiliations:** 1 Institut de Biologia Evolutiva (UPF-CSIC), Universitat Pompeu Fabra, Dr. Aiguader, 88 08003 Barcelona, Catalonia Spain; 2 Estonian Biocentre, Institute of Genomics, University of Tartu, 51010 Tartu, Estonia; 3 Department of Biology, University of Padova, 35131 Padova, Italy; 4 The Wellcome Sanger Institute, Wellcome Genome Campus, Hinxton, Cambridgeshire, CB10 1SA UK; 5 Bioinformatics Studies, ESCI-UPF, Barcelona, Catalonia Spain

**Keywords:** Positive selection, Selective sweeps, Human population genetics, Genomics, Admixture, African populations, Ethiopia, West Asia

## Abstract

**Background:**

In the process of adaptation of humans to their environment, positive or adaptive selection has played a main role. Positive selection has, however, been under-studied in African populations, despite their diversity and importance for understanding human history.

**Results:**

Here, we have used 119 available whole-genome sequences from five Ethiopian populations (Amhara, Oromo, Somali, Wolayta and Gumuz) to investigate the modes and targets of positive selection in this part of the world. The site frequency spectrum-based test SFselect was applied to idfentify a wide range of events of selection (old and recent), and the haplotype-based statistic integrated haplotype score to detect more recent events, in each case with evaluation of the significance of candidate signals by extensive simulations. Additional insights were provided by considering admixture proportions and functional categories of genes. We identified both individual loci that are likely targets of classic sweeps and groups of genes that may have experienced polygenic adaptation. We found population-specific as well as shared signals of selection, with folate metabolism and the related ultraviolet response and skin pigmentation standing out as a shared pathway, perhaps as a response to the high levels of ultraviolet irradiation, and in addition strong signals in genes such as *IFNA*, *MRC1*, immunoglobulins and T-cell receptors which contribute to defend against pathogens.

**Conclusions:**

Signals of positive selection were detected in Ethiopian populations revealing novel adaptations in East Africa, and abundant targets for functional follow-up.

## Background

Genetic and archaeological data demonstrate that Africa is the origin of anatomically modern humans [1–4], and that populations outside Africa derive from an Out-of-Africa (OoA) migration some 60,000 years ago [5–9]. African populations are genetically more diverse than any other human population, holding the highest amount of genetic variation, low linkage disequilibrium (LD), and deep population structure [10–13]. They also carry high cultural and phenotypic diversity, speak almost one-third of the world’s languages [14], live in a wide variety of environments including deserts, tropical rainforests and mountain highlands, and follow many subsistence strategies, including pastoralism, agriculture and hunter-gathering [15]. However, African populations are underrepresented in big genetic projects such as the HGDP [16], 1000 Genomes Project [17] and HapMap [18]. Consequently, not only is our understanding of the evolutionary processes that shape human diversity and adaptation limited, but also medical studies are prone to falter when African populations are included, due either to the fact that the single nucleotide polymorphisms (SNP) used are ascertained mainly in Eurasian populations, or to the lower LD found in all African populations [13]. Additional African-specific studies are needed to counterbalance this historical bias [13, 19].

Ethiopian populations lie geographically near a possible embarkation point of the OoA migration [20, 21], exhibit high linguistic diversity encompassing three branches of the Afroasiatic language family (Omotic, Semitic, Cushitic) and also the Nilotic language family, and inhabit environments from lowland to highland. Previous genotyping studies have found a strong match between linguistic and genetic structures, and revealed admixture between Ethiopian (principally Afroasiatic) and OoA populations (most likely from West Asia) around 2600 years ago, contributing about half of the ancestry of some present-day populations in what has been called a “back to Africa” migration [11, 22, 23]. A 4500-year old ancient Ethiopian fossil, Mota, does not show this West Asian backflow [24], and provides direct insights into the earlier genetic make-up.

Because African populations have adapted to a variety of environments and subsistence strategies, it is crucial to conduct natural selection studies in order to observe how selective pressures shaped their genomes and understand both our evolutionary history as a species and the population-specific local adaptations to these circumstances. Given the diverse features of African populations, we could expect to find a considerable number of signals of local adaptation. A number of approaches have been established to detect positive selection [25–27], and a few signals of adaptive selection in Africans have been reported. Some of the most well-known cases involve malaria resistance, driven by genes such as Glucose-6-phosphate dehydrogenase (*G6PD*) and the Duffy antigen protein [28]. There is also evidence of high-altitude adaptation in Ethiopians living in the highlands, as well of recent positive selection for lactase persistence in eastern African pastoralists [9, 29–31]. However, genome-wide analyses of adaptive selection footprints have often reported fewer signals in Africans than in OoA populations [32–34], or failed to find adaptive selection in Africans, some arguing that neutral simulations demonstrate that the tails of the empirical distributions contain mainly false positive signals [35], meaning that demographic events (bottlenecks, population structure and expansions), rather than selection, dominate the results [36]. Thus African populations offer a challenge in recognizing events of adaptive selection in the genome.

In addition, the power and false-positive rates of positive selection tests in recently-admixed populations have only been addressed in a few studies. In a study of African-Americans using real and simulated genetic data, recent admixture did not result in an increase of false positive rates for site frequency spectrum-based tests, but in general the power decreased [37]. In contrast, in some cases when the selective pressure was very strong, studying the admixed population could provide more power to detect selection than the ancestral population because the signature of derived alleles around the fixed selected site was lost in the ancestral population, but admixture made them polymorphic again producing a signature that is easier to detect. Studies with Latin American [38], Tibetan [39], Malagasy [40] and South Asian [41] populations have found potential admixture-mediated adaptive regions using this methodology, although some controversy exists since another Latin American study did not find evidence of directional selection after admixture [42].

These examples reveal that detecting positive selection is far from trivial. Most positive selection tests assume a simple model of a hard sweep, where a mutation arises and spreads rapidly in a population until fixation, carrying the adjacent neutral variation with it [43]. However, the relative importance of hard versus soft sweeps in explaining the adaptation of different human populations is debated, and all forms of selection need to be considered [17, 44–47].

Here, we analyse previously-generated whole-genome sequences of 119 Ethiopians from five different populations [7] covering a wide geographical range and belonging to four different linguistic groups (Nilotic, Omotic, Cushitic and Semitic) (Table 1 and Supplementary Fig. 1 in Additional File 1). We provide new information about the adaptive processes that these populations have undergone by first detecting the regions of the genome that have been selected, and then interpreting the biological meaning and context of these adaptations.

**Table 1:** Ethiopian populations, linguistic families and sample sizes included in the study

Population	Linguistic family	Linguistic subfamily	Number of samples
Amhara	Afroasiatic	Semitic	24
Oromo	Afroasiatic	Cushitic	24
Somali	Afroasiatic	Cushitic	24
Wolayta	Afroasiatic	Omotic	23
Gumuz	Nilo-Saharan	Nilo-Saharan	24

## Results

### SFselect selection analysis

In order to detect selective sweeps, we first analysed the data using the site frequency spectrum-based test SFselect [48] to identify old events of selection in the five Ethiopian populations (Supplementary Fig. 2, Additional File 1). This approach generates a score for each 30 kilobase (kb) window in the genome. We assessed the statistical significance of the scores by defining a critical value of the test, after performing extensive neutral simulations (see methods), as corresponding to the 99.99th percentile of the neutral distribution (see Methods); the threshold is different for each population. Our simulations were based on a three-population demographic model representing Africans, Europeans and Asians [49], adding an admixture event between Africans and Europeans. We calculated two different thresholds, one for an unadmixed African population (here, the Gumuz) and the second for an admixed African population (here, the four Afroasiatic populations) (Supplementary Fig. 3 in Additional File 1 and Supplementary Table 1 in Additional file 2). After applying the relevant threshold to each of the five populations, we obtained windows considered as putative candidates for adaptive selection. The number of significant windows is shown in Table 2a.

**Table 2:** Number of shared 30 kb windows under selection between East African populations. Significant windows for each population (n) were selected after applying the 99.99 thresholds calculated after the neutral simulations. **A**) SFselect **B**) iHS

A	Amhara (*n* = 207)	Oromo (*n* = 220)	Somali (*n* = 158)	Wolayta (*n* = 192)	Gumuz (*n* = 182)
Amhara		79	58	72	41
Oromo			54	67	40
Somali				54	38
Wolayta					37
**B**	Amhara (*n* = 35)	Oromo (*n* = 41)	Somali (*n* = 66)	Wolayta (*n* = 54)	Gumuz (*n* = 70)
Amhara		7	6	8	5
Oromo			7	11	5
Somali				4	4
Wolayta					6

**Fig. 1:**
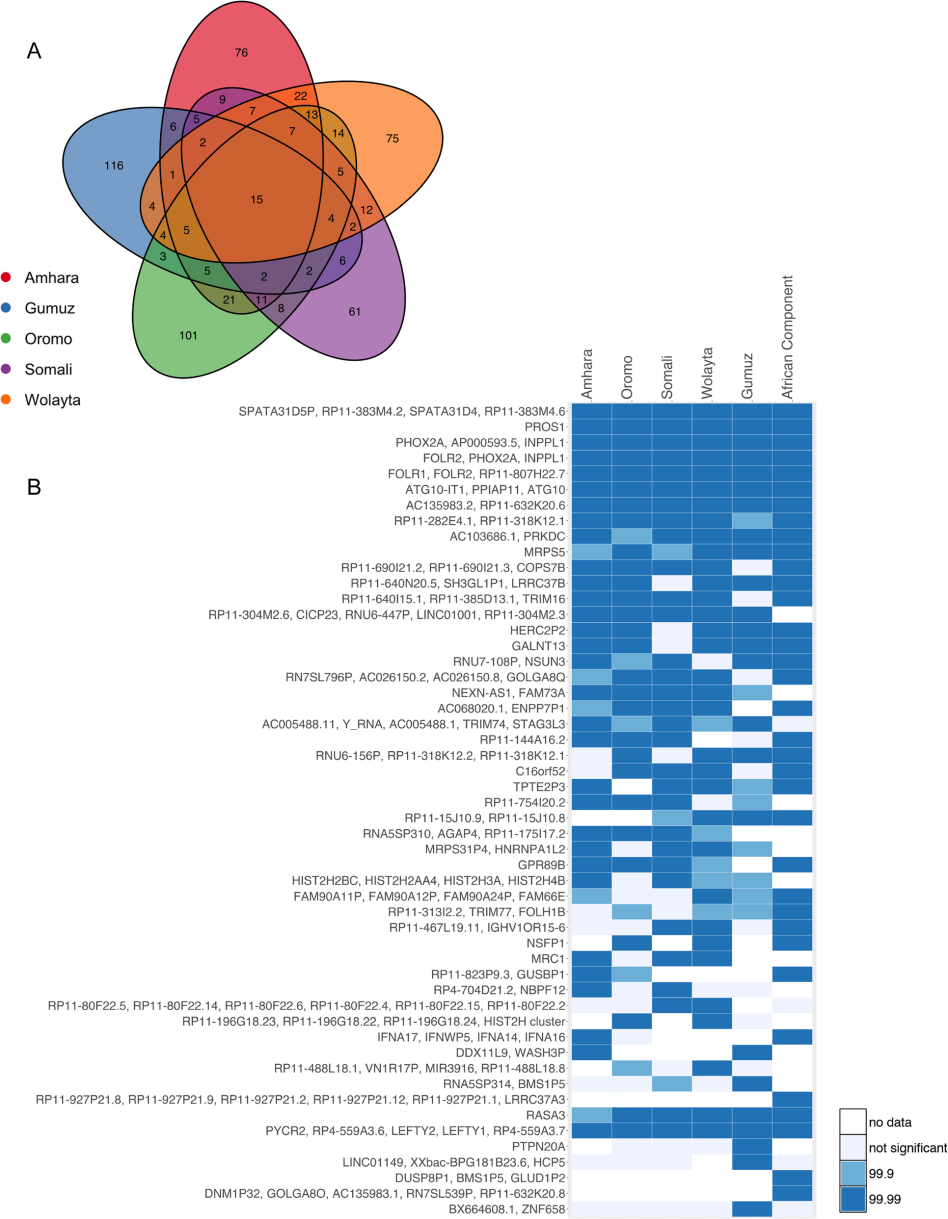
Shared signals of positive selection between populations detected with SFselect. A) Venn diagram of the number of windows above the 99.99th percentile threshold shared between populations. B) Genes in the top 20 windows above the 99.99th percentile threshold by population. Each row represents a window and colours indicate the population corresponding significance

To interpret these windows, we annotated the protein-coding genes that intersected them (Supplementary Table 2, Additional file 2). Many of the signals were shared between Afroasiatic populations (Table 2a and Fig. 1a), as expected from their genetic similarity and shared environment (see Additional File 3 and Supplementary Figs. 4 and 5 in Additional File 1 for a short demographic analysis of the studied populations). The Amhara and Oromo populations shared the highest number of signals (79), whereas the Gumuz shared the least (from 37 to 41). We found many examples of shared signals of selection by all five East African populations (Supplementary Table 3 in Additional file 2 and Fig. 1). We discuss illustrative examples of shared and population-specific signals here, and further examples in the Additional File 3.

**Fig. 2:**
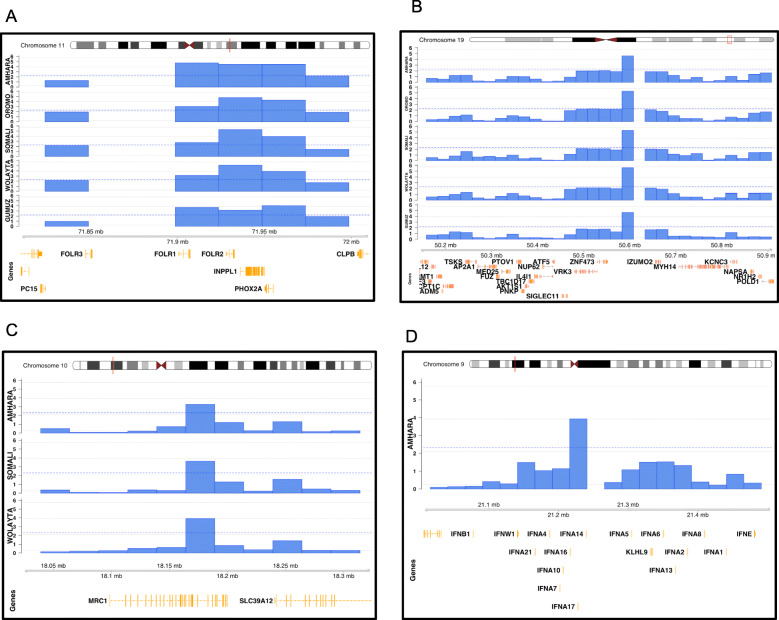
Genomic context of some of the significant regions from the SFselect analysis. Each bar represents to a 30 kb window, the y-axis corresponds to the SFselect score, blue dotted lines indicate the 99.99th percentile threshold obtained from extensive neutral simulations

One of the top-scoring windows in all populations (Amhara 4.8, Oromo 4.7, Somali 5.2, Wolayta 5.1, Gumuz 3.7) contains genes including *FOLR1* and *FOLR2* (Fig. 2a), members of the folic acid receptor family. Members of this gene family bind folic acid and its reduced derivatives, and transport 5-methyltetrahydrofolate into cells. The gene products are secreted proteins that either anchor to membranes via a glycosyl-phosphatidylinositol linkage or exist in a soluble form. Mutations in these genes have been associated with neurodegeneration due to cerebral folate transport deficiency; supplementation of folic acid is usually recommended for pregnant women to avoid neural tube defects during foetal development [50]. Folate is also essential in DNA synthesis, survival and growth of the malaria parasite, so antifolate antimalarial drugs are widely used in the treatment of malaria [51]. To our knowledge, this is the first study that finds this gene cluster to be under selection. The fact that we found this window under selection in all populations, together with the important functions of these genes especially during development, indicates that these genes have probably played a pivotal role during the evolutionary history of East Africans and possibly in general within the human species. We discovered, and discuss below, other selection signals related directly or indirectly to folic acid metabolism.

Another example of a top-scoring window among all five populations does not directly overlap with any gene, but the very strong signal lies downstream of the gene *ZNF473* (Fig. 2b and Supplementary Table 2 in Additional file 2). This is an interesting region since it has been described as under long-term balancing selection in African populations, and that has been recently targeted by positive selection in Eurasian populations [52].

A signal shared among the Amhara, Somali and Wolayta populations contains *MRC1* (Supplementary Table 2a, c, d in Additional file 2 and Fig. 2c). *MRC1* (also known as *CD206*) encodes a mannose receptor that is part of the C-type lectin superfamily and plays important roles in both adaptive and innate immune systems such as clearance of endogenous molecules and antigen presentation [53]. MRC1 is an endocytic receptor that can bind to numerous endogenous and exogenous molecules and is mainly expressed in macrophages, dendritic cells and nonvascular epithelium [54]. Numerous studies have shown that the C-type lectin-like domain (CTLD) of MRC1 can bind to viruses (HIV, Dengue, HBV), fungi (*Candida albicans*) and bacteria (*Mycobacterium tuberculosis*) [55]. It has also been shown that MRC1 can internalize antigens that can then be processed for cross-presentation in antigen-presenting cells [56] and that it directly interacts with and inhibits CD45 on the T-cell surface resulting in impaired cytotoxic activity of T-cells and antigen-specific T-cell tolerance [57]. This inhibitory effect of T cells by MRC1 has been proposed as a possible therapeutic strategy to downregulate the excessive immune response of autoimmune diseases. In fact, variants in *MRC1* have been associated with asthma [58] and sarcoidosis [59]. In addition, variants of *MRC1* have been associated with susceptibility to leprosy in Vietnamese and Brazilian patients [60] and to pulmonary tuberculosis in Chinese patients [61]. This example introduces a second recurring theme, of selection on defence-related genes, which will be encountered further below.

We also detected population-specific signals of positive selection, and a particularly strong signal was found in the Amhara population, where the 30 kb window containing *IFNA14*, *IFNA16* and *IFNA17* showed a very high and statistically significant SFselect score of 3.9. These genes are members of the Interferon Alpha gene family (Fig. 2d and Supplementary Table 2a in Additional file 2); Interferon Alpha is produced in virus-infected leukocytes and has antiviral activity. It has been shown in vitro that *IFNA17* is three times more efficient against Hepatitis C than *IFNA2A*, which is the most effective current treatment [62]. Moreover, polymorphisms in *IFNA17* have been associated with a 3.6-fold increased risk for Crimean-Congo Haemorrhagic Fever development [63]. These interferon genes provide further examples of selection on likely defence against pathogens. Other categories of population-specific signal are discussed in the Additional File 3.

### iHS selection analysis captures recent events of selection

We next used the linkage-disequilibrium-based test iHS [64] to detect recent events of selection in the five Ethiopian populations (Supplementary Fig. 6 in Additional File 1). We analysed mean iHS scores in windows of 30 kb that passed the critical value defined after performing extensive neutral simulations (see Methods). We set a restrictive threshold at the 99.99th percentile of the neutral distribution and only signals that passed this threshold were considered as candidates for adaptive selection (Supplementary Table 1 in Additional file 2 and Supplementary Fig. 3 in Additional File 1).

**Fig. 3:**
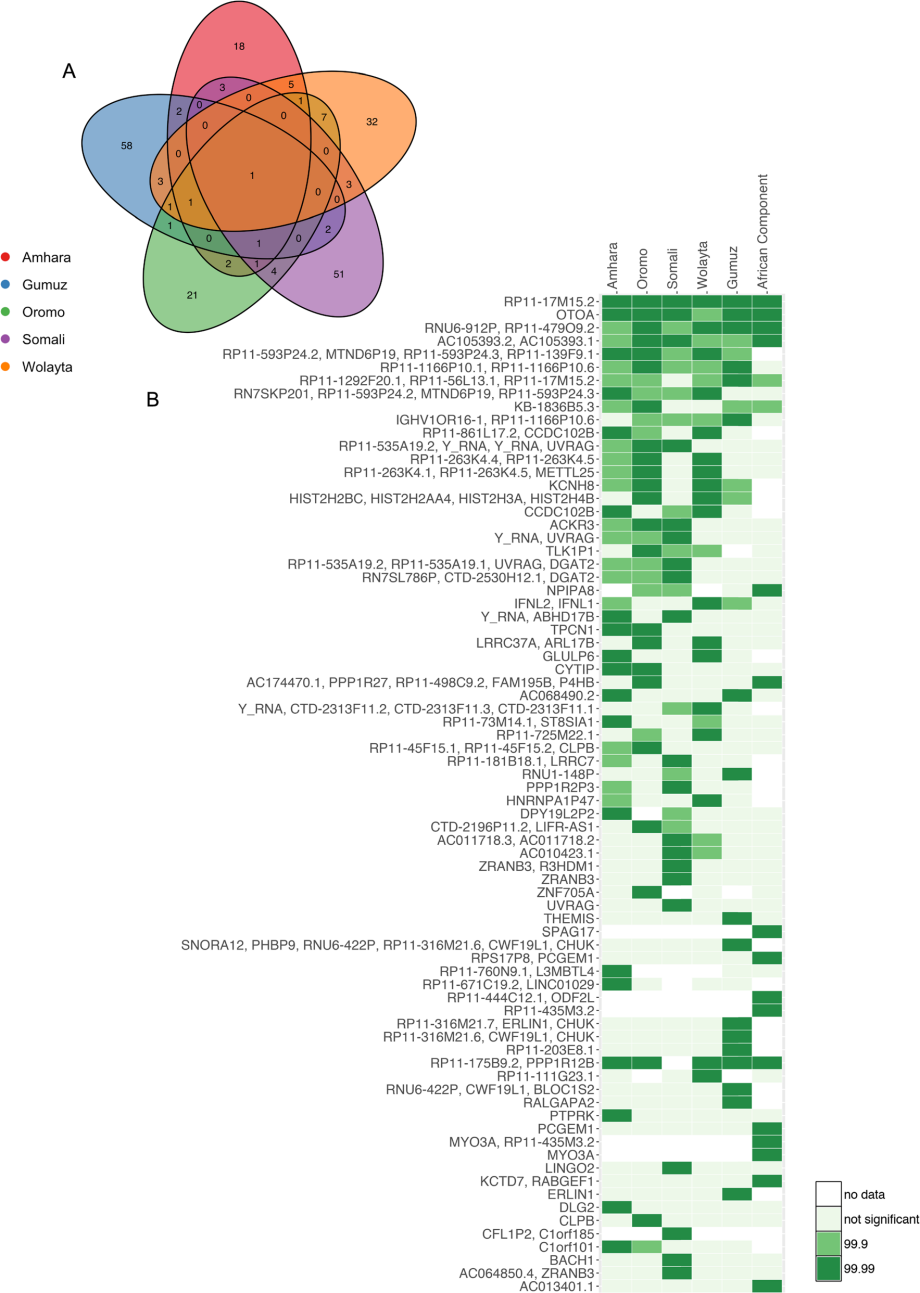
Shared signals of positive selection between populations detected with iHS. A) Venn diagram of the number of windows above the 99.99th percentile threshold shared between populations. B) Genes in the top 20 windows above the 99.99th percentile threshold by population. Each row represents a window and colours indicate the population corresponding significance

The number of significant windows per population was low, and the five populations shared some windows under recent positive selection (Table 2b, Supplementary Table 4 in Additional file 2 and Fig. 3a). Amhara and Oromo shared the highest number of windows (12 each) with Wolayta, while the lowest number of window shared (four each) was between Somali and Wolayta, and Somali and Gumuz. The Gumuz in general shared the lowest number of windows with the rest of the populations. The lower numbers of significant windows and shared windows from the iHS analysis compared with the SFselect analysis could be because the populations split quite recently, so there has been little time for selection signals to build up.

**Fig. 4:**
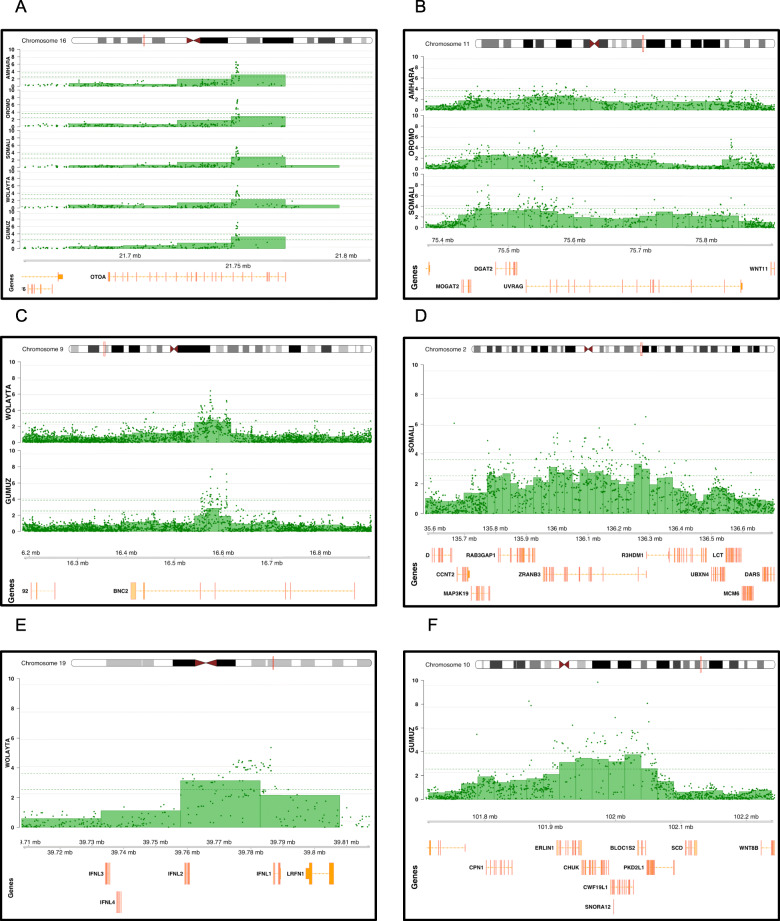
Genomic context of some of the significant regions from the iHS analysis. Each bar represents the absolute mean iHS of a 30 kb window, each green dot corresponds to the –log10(*p*-value) of a single variant. The y-axis corresponds both to the normalized mean absolute iHS score per window and the –log10(*p*-value) per variant. Green dotted lines indicate the 99.99th percentile thresholds of the absolute mean iHS per 30 kb windows (lower line) and the –log10(*p*-value) per variant (upper line) obtained from extensive neutral simulations

Although the intersection of signals between all five populations is modest, we do find some strong shared signals (Supplementary Table 4 in Additional file 2). *OTOA* (otoancorin) shows high and significant mean iHS scores in all populations (except Wolayta, which is close) and variants with significant *p*-values. Specifically, one of the top variants in all populations (rs370153558) show *p*-values of 1.8 × 10^− 6^, 5 × 10^− 8^, 3.4 × 10^− 5^, 1 × 10^− 6^, 9 × 10^− 8^ for Amhara, Oromo, Somali, Wolayta and Gumuz respectively (Fig. 4a). All the variants in the *OTOA* gene found under strong selection lie in the intron 21. The protein encoded by *OTOA* is expressed on the apical surface of epithelial cells in the sensory organs of the inner ear. Mutations in *OTOA* have been found in Palestinians and Pakistanis to be causative for autosomal recessive deafness 22 [65, 66]. Hearing is a rapidly-evolving phenotype in humans [67] and thus the likely target of selection here.

Several examples of selection signals were related to ultraviolet (UV) protection or skin pigmentation, and thus indirectly to the folic acid metabolism discussed above. One of these was *UVRAG*, where we find a signal in all Afroasiatic populations. The strongest signal is found in the Oromo and Somali with mean iHS scores of 2.6 and 3.4 in the region and specific intronic variants such as rs10899132 (Somali *p* = 10^− 6^, Oromo p = 10^− 4^) are found although no clear functional predictions are yet described (Supplementary Table 5 in Additional file 2 and Fig. 4b). The Amhara also show significant mean iHS scores in other windows containing *UVRAG* (mean iHS score of 2.65 and rs7117696 variant with *p* = 5 × 10^− 5^). The lack of a signal from SFselect in this region of the genome also supports the idea of recent selection. *UVRAG* plays an essential role in protecting cells from UV-induced DNA damage by activating the nucleotide excision repair pathway [68]. In addition, it acts as an autophagic tumour suppressor that is mutated in common human cancers [69].

A second candidate, shared between Wolayta and Gumuz, is *BNC2* with mean iHS scores per window 2.79 and 2.86 (Fig. 4c) and specific variants such as rs113571602 with significant *p*-values (4 × 10^− 7^ and 2 × 10^− 7^ respectively). Again, all the highest scoring variants fall in an intron of *BNC2*, pointing towards a putative regulatory change of gene expression. This gene codes for a DNA-binding zinc-finger protein that acts as an mRNA-processing enzyme and a transcription factor [70]. It is expressed in melanocytes and keratinocytes and variants have been associated with skin colour, where higher expression levels correspond to darker skin [71]. Interestingly, *BNC2* has been found to lie in an adaptive introgressed region from Neanderthals to Europeans [72], but the signal of selection in Ethiopia lies outside the reported introgressed region.

The third example in this category is found in a region containing *ZRANB3* with statistically significant mean iHS scores of 3.32 and variants with significant *p*-values such as rs11892059 (*p* = 1.8 × 10^− 6^) (Supplementary Table 5c in Additional file 2, Fig. 4d). ZRANB3 is an annealing helicase, fork remodeller and structure-specific nuclease; its deficiency can cause genome instability and hypersensitivity to diverse DNA damaging agents such as UV radiation [73]. This region has previously been reported as a putative selection candidate in the Maasai population [74] but the authors did not link the signal to adaptation related to UV radiation because this variant is in linkage disequilibrium with the well-known lactase (*LCT*) gene which has many times been reported to be under selection in several populations [29, 75]. In Ethiopians, the signal is clearly in *ZRANB3* and not in *LCT*. *ZRANB3* has also been found under selection among black Tibetan wild boars, providing more evidence for its important function to maintain genomic stability against the high UV radiation found in the Tibetan Plateau [76].

Many examples of selection signals related to defence were also found. Among these was a window in the Wolayta showing a statistically significant mean iHS of 3.14 and many variants with *p* < 10^− 5^ in the upstream region of *IFNL1* and the intergenic location between *IFNL1* and *IFNL2*. Signals in the 99.9th percentile are also found in the Amhara and Gumuz (Supplementary Table 5d in Additional file 2 and Fig. 4e). The Interferon-λ family or type III IFNs has three members (*IFNL1*, *IFNL2*, *IFNL3*). These genes play a critical role in antiviral, antiproliferative, antitumor and immune responses [77]. These responses often overlap with IFN-α functions such as MHC class I antigen expression and induction of antiviral cascades. Some of the antiviral activities of INFLs target hepatitis B and C virus, cytomegalovirus, influenza A virus, coronaviruses, encephalomyocarditis virus, intestinal infection viruses (noroviruses and rotaviruses) and human immunodeficiency virus by inducing the expression of antiviral proteins by the infected cells [78]. Clinical trials against hepatitis C virus have tested PEGylated IFNL1 and showed a better or equal effectiveness than PEGylated IFN-α with less extrahepatic adverse effects [79]. Since humans are very frequently exposed to viruses of low pathogenicity, and IFN-λ mostly targets mucosal epithelial cells, the function of type III IFNs could be to protect from infections without triggering the severe inflammation and tissue damage that type I IFNs often produce in the long term [80]. Hence IFN-λ could have been a good target of local adaptation to newly encountered pathogens. Additional signals related to defence in *TPCN1, CHUK, THEMIS* and *TRAV* are discussed in the Additional File 3.

Finally, a signal specific to Gumuz was found in *PKD2L1* (Fig. 4f), with high iHS scoring variants such as rs74154621 and rs74154622 (iHS score *p* < 10^− 8^) that are both whole blood eQTLs with a normalized effect size of − 0.669 and p < 10^− 4^ according to the GTEx portal. We also find several non-synonymous changes with a high derived allele frequency in the Gumuz (rs17112895 and rs7909153 both at a frequency of 0.70). *PKD2L1* belongs to the TRPP subfamily of ion channels that are characterised by large extracellular domains [81]. It is expressed in a subset of taste receptor cells in specific taste areas in mice [82] and has been identified as a candidate for sour taste in mammals [83]. In humans, two patients with sour taste ageusia have been reported and neither had detectable *PKD2L1* transcripts, indicating a potential role of *PKD2L1* in human sour taste [84]. Sour taste is one of the five basic tastes and although other tastes have a clear evolutionary purpose (sweet indicates carbohydrate rich food, salty taste sodium, bitter potentially poisonous and umami protein rich), sour tasting remains unexplored in humans. One of the main hypotheses of the evolutionary sour tasting function is that it could warn against the acidic ingestion of rotten or immature fruit [85]. Further signals of selection from other functional categories such as skin pigmentation (*BLOC1S2*) and one in an RNA gene (*NSUN3*), were also detected (Additional File 3).

### Effect of admixture on detecting ancient and recent selection

The power and false-positive rates of positive selection tests in admixed populations have only been addressed in a few studies [9, 37–40]. To provide further support for our selection analyses, we have investigated whether similar results could be obtained without the West Asian ancestry genetic component among the Afroasiatic populations. For that purpose, we masked the West Asian component from our data, keeping only the East African component (see Methods). Given that, on average, almost half of the genome was masked by this procedure, we merged all four Afroasiatic populations in a single meta-population and re-ran the positive selection tests used previously. Principal Component Analysis (PCA) of the retained East African component confirmed the high similarity between the East African component of the Afroasiatic populations, supporting the combined meta-analysis of all the individuals (Supplementary Fig. 7 in Additional File 1).

The comparisons between the top 20 signals of the SFselect analysis between each single population and the merged East African component show a high similarity between the two analyses (Fig. 1b). In contrast, the overlaps of the iHS analyses were not as strong (Fig. 3b). This last result could be due because of the breaking down of the Ethiopian haplotypes by the ancestry switches that occurred after the West Asian admixture in the area or because of the nature of iHS that detects recent selection more likely to be specific to each population.

### Enrichment of west Asian ancestry in windows under selection

The masked West Asian component measures the proportion of West Asian ancestry in each population (Table 3). The Amhara and Oromo populations have the highest amount (54 and 51%, respectively), Wolayta and Somali show 43 and 44%, respectively, while in contrast the Gumuz show the low amount of 0.7%. These values agree with previous estimates [7]. To detect for any enrichment of West Asian ancestry in windows under selection, the same calculation of the proportion of the West Asian component was performed among regions under positive selection (99.99th percentile after neutral simulations and 1% extreme scores) for all populations, for both iHS and SFselect. This analysis revealed a general increase of West Asian ancestry among the regions putatively under selection found with both the SFselect and iHS tests, with similar percentages for the two tests (Table 3). A resampling analysis shows that the difference is highly significant (*p* < 10^− 5^, see Methods): there is thus an overall enrichment of West Asian ancestry in regions under selection. It is worth mentioning that this enrichment of West Asian ancestry is not a source of false positive signals in our analysis given the results obtained when we analysed the effect of admixture on detecting adaptive selection (see above).

**Table 3:** Average West Asian ancestry proportions for each population at the genome level and among the significant windows under selection from SFselect and iHS analysis. Significant windows for each population (n) were selected after applying the 99.99 thresholds calculated after the neutral simulations

	Whole Genome	SFselect	iHS
Amhara	0.54	0.60	0.67
Oromo	0.51	0.56	0.62
Somali	0.45	0.49	0.61
Wolayta	0.43	0.49	0.56
Gumuz	0.07	0.10	0.15

### Unbalanced ancestry regions

Previous studies have used ancestral component proportions to detect regions with a strong ancestry imbalance that could potentially have positive or negative effects on the fitness of admixed populations [41]. The admixture event between Ethiopian and West Asian populations is dated to 2500–3000 years ago [7, 11], meaning that under a neutral model, we would expect the percentage of the West Asian ancestry component to be evenly distributed across the genome. Therefore we report regions with significant deviations from the expected distribution of West Asian component in several populations that could be candidates of adaptation.

**Fig. 5:**
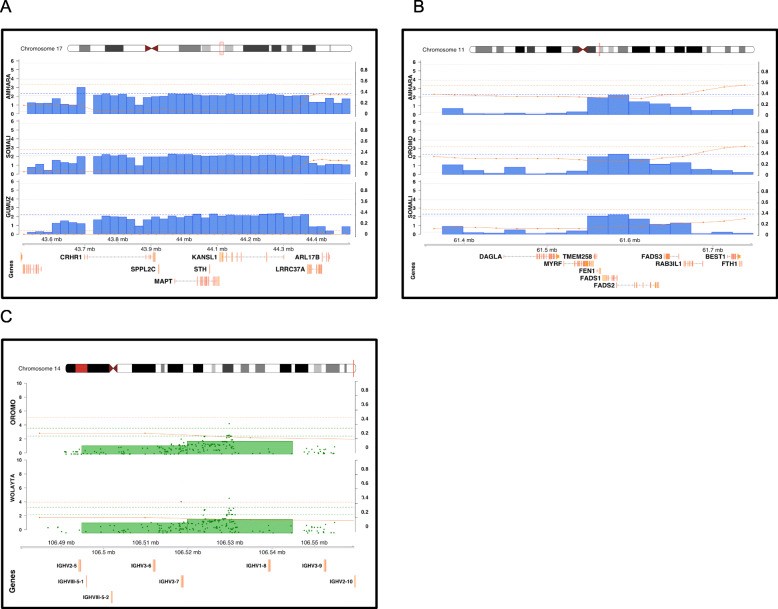
Genomic context of some of the regions with a strong deviation of West Asian ancestry. Orange solid lines represent the mean West Asian ancestry of the region and orange dotted lines the genome wide mean of West Asian ancestry specific of the population. Plots A and B also include in blue SFselect scores per 30 kb windows, left y-axis corresponds to SFselect scores and right y-axis the proportion of West Asian ancestry. Plot C includes in green absolute mean iHS per 30 kb region and green dots –log10(*p*-values) per single variant

A good example is a long stretch of chromosome 17 with an extreme 95% of African ancestry spanning more than 0.5 Megabases (Mb) in all Afroasiatic populations. Moreover, in this region we find the *CRHR1* (Corticotrophin Releasing Hormone Receptor 1) gene with a high SFselect score of 3 (significant after simulations) in the Amhara, and 2.22 and 2.1 in the Somali and Gumuz, respectively (close to significance). There are other genes in the region such as *KANSL1* and *MAPT* (Fig. 5a). We also find in all Afroasiatic populations, except Wolayta, an excess of African ancestry in windows under selection containing among other genes *FADS1* and *FADS2*, two enzymes that participate in the omega-3 and omega-6 biosynthesis and found to be under positive selection in other human populations (Fig. 5b) [86, 87]. In Oromo and Wolayta, high African ancestry (77 and 81% respectively) and high iHS scores (top scoring SNPs with *p* < 10^− 4^) were found around the immunoglobulin heavy variable 1–8 genes (*IGHV1–8*), central to defence (Fig. 5c).

### Signatures of polygenic adaptation through functional enrichment analysis

Functional enrichment analysis can be used to understand the biological functions of groups of genes, in this case those that have putatively been under positive selection. For this analysis, we listed the genes contained in windows with scores higher than the empirical 99.5 percentile, either for SFselect or mean iHS. We relaxed the thresholds of significance since we are trying to detect loci contributing to polygenic selection and a biological term was considered significant if the *p*-value after a Benjamini-Hochbert correction was below an alpha value of 0.05 (Table 4). Details of the significant terms and associated genes in each population, and selection tests, can be found in the Additional file 3. Many of the biological categories significant in all populations are related to immune responses and defence (Table 4). Folate metabolism is also a recurrent function found in many populations, as well as for calcium homeostasis related functions. Finally, muscle development function also appears in several populations.

**Table 4:** Signatures of polygenic adaptation through functional enrichment analysis. We have listed the most relevant terms of the analysis. The two lists of genes used for the analysis were taken from the significant windows under putative positive selection for SFselect and iHS. The genes used for this analysis are listing the genes with significant SFselect or iHS scores. A biological term was considered significant if the *p*-value after a Benjamini-Hochbert (BH) correction was below an alpha value of 0.05

Term	Population	P-value (BH)	Test
Response to virus	Amhara	0.006	SFselect
RNA surveillance	Somali	0.005	SFselect
Regulation of viral process	Wolayta	0.026	SFselect
Type I interferon binding	Amhara	0.018	SFselect
Type I interferon production	Gumuz	0.020	SFselect
Positive regulation of interferon-gamma production	Gumuz	0.027	iHS
B-cell activation and regulation of immunoglobulin production	Amhara, Somali	0.017; 0.01	SFselect
Regulation of immunoglobulin production	Amhara	0.018	SFselect
Hepatitis B	Amhara	0.03	SFselect
Tuberculosis	Amhara	0.047	SFselect
Measles	Amhara	0.04	SFselect
Leishmaniasis	Gumuz	0.049	iHS
Lupus erythematous	Somali, Wolayta, Gumuz	0.0009; 0.02; 0.026	iHS
Folic acid containing compound metabolic process	Amhara, Somali, Wolayta	0.013; 0.02; 0.02	SFselect
Folic acid metabolic process	Amhara, Wolayta	0.019; 0.01	SFselect
Metabolism of folate	Amhara	0.017	SFselect
Pterines and folate biosynthesis	Amhara	0.02	SFselect
Cellular response to UV-B	Amhara, Somali	0.002; 0.0019	SFselect
Cellular response to UV	Amhara, Somali	0.018; 0.0027	SFselect
Cellular response to radiation	Amhara, Somali	0.01; 0.001	SFselect
Cellular response to vitamin D	Amhara	0.02	SFselect
Bone mineralization	Somali	0.03	SFselect
Osteoclast differentiation	Wolayta	0.03	SFselect
Negative regulation of cardiac muscle tissue development	Gumuz	0.02	SFselect
Negative regulation of striated muscle tissue development	Gumuz	0.037	SFselect
Muscle fibre development	Somali	0.036	iHS

All in all, the enrichment analysis reinforced our previous analyses of selection, again highlighting several of the main adaptations that Ethiopian populations have undergone.

## Discussion

In this study, we have found new gene candidates under adaptive selection in populations from East Africa. We have been able, by performing extensive simulations, to assess the significance of our candidate adaptive selection signals. We have provided evidence for both old and recent selective sweeps, and both shared and population-specific signals of selection, while accounting for any effect of admixture. Our work has also highlighted the genetic similarity among Afroasiatic Ethiopian populations since many of the old signals of selection are shared between them.

Selection analysis in recently admixed populations is of special interest as the adaptation process may maintain pre-admixture adaptations or use one of the components as the genetic background for new adaptations. It is thus of interest to compare the more ancient (likely pre-admixture) and more recent (post-admixture and population specific) adaptations. The site-frequency-based test SFselect captures ancient and shared selection events before the gene flow from West Asia into Africa and thus before the admixture of the West Asian and the East African components. Conversely, iHS captures recent selection that probably happened after admixture, and that is often population-specific either for the Nilotic (Gumuz) or Afroasiatic populations (Amhara, Oromo, Wolayta, Somali).

We have found that folate metabolism appears to have been crucial for Ethiopian populations, a trait that is new as an adaptation [88]. Specifically, we have identified the genes *FOLR1, FOLR2* and *DHFRL1* (see Additional File 3) as candidates of adaptive selection, while the functional enrichment analysis also highlighted folate metabolism as a main function potentially under selection, and many genes related to skin pigmentation or UV protection were picked out. Folate is crucial for DNA biosynthesis, methylation and repair and its deficiency can cause fatal birth defects and hence can directly affect reproductive success. Sufficient folate is associated with a 72% reduced risk of neural tube defects [50] and it is known that folate deficiency severely challenges the nucleotide excision repair mechanism needed to remove UV induced DNA photoproducts [89]. Ethiopia experiences very high ultraviolet radiation, which has consequences that include severe DNA damage and impaired genome integrity. It has been hypothesized that under high UVB and UVA radiation, dark skin pigmentation has been selected in order to avoid folate photolysis (the “vitamin D-folate hypothesis”) [90, 91]. Among the recent selective sweeps were many on genes involved in UV radiation response and pigmentation. In the Afroasiatic populations, we have found a region containing the *UVRAG* gene that activates the nucleotide excision repair pathway when there is UV-induced damage in cells. We have also found as selection candidates *BNC2* (among Amhara, Oromo, Wolayta and Gumuz) whose high expression is associated with dark skin colour, and *BLOC1S2,* encoding a subunit of the complex BLOC-1 that produces strong pigmentation phenotypes in mice and Hermansky-Pudlak syndrome in humans and also many functional enrichment categories related to UV responses. In addition, we have found *ZRANB3* in the Somali population, where deficiency causes genome instability and hypersensitivity to DNA damaging including UV radiation and has been found to be under selection in black boars from the Tibetan plateau (an area also challenged with a high UV radiation) [76]. Thus, there is strong evidence in our study pointing towards folate and pigmentation related adaptations.

The environmental changes and migrations that humans have often experienced have made immunological adaptations a key process during human evolution. Our study gives further insights into these immune-related adaptations in East Africa where the major causes of death are due to infections (HIV, tuberculosis, malaria and other acute lower respiratory infections). For example, we have found in Amhara a region containing *IFNA* genes that encode for interferon alpha (pivotal for antiviral responses) and a region that the Amhara, Somali and Wolayta share in common containing *MRC1* (an endocytic receptor involved in adaptive and innate immune responses). Most importantly, we have found in the Gumuz population regions under potentially recent adaptive selection containing genes belonging to the immunoglobulin heavy constant and variable chains and to the T-cell receptor alpha variable locus.

## Conclusions

We used two positive selection methods to detect signals of adaptation in the genomes of five Ethiopian populations. Although we have been able to highlight potentially adaptive regions through computational methods and elaborated on the possible biological implications that could have been pivotal for adaptation in East Africa, this is just a first step towards a better understanding of human adaptive evolution. Our work provides the foundation for further functional studies that are necessary to fully understand the adaptive phenotype behind the footprints of positive selection in Ethiopian genomes.

## Methods

### Data

The dataset comprised five East African populations (Amhara, Oromo, Somali, Wolayta and Gumuz) with 24 individuals each from Pagani et al. 2015. One Wolayta individual was excluded from all subsequent analysis due to a high degree of relatedness (data not shown). Additional samples from the 1000 Genomes Project [17] and a set of 100 Egyptian samples also from Pagani et al. 2015 were included in PCA and ADMIXTURE analyses. The genome assembly of the data is GRCh37 (hg19). A summary of the dataset is shown in Table 1.

### PCA and ADMIXTURE

The Principal Component Analysis was performed with *smartpca* from the Eigensoft 6.0.1 software [92]. All individuals from the dataset were used to perform the worldwide PCA. For the local PCA, all Ethiopians were included, plus a random subset of 24 West African Yoruba (YRI) and 24 European (CEU) individuals from 1000 Genomes Project [17]. We applied a general filter requiring minor allele frequency higher than 0.05. The PCA of the West Asian masked samples was done with the lsqproject mode that is suitable when the samples have large amounts of missing data.

Population structure analysis was performed with the ADMIXTURE software [93] on a reduced set of 13 populations, 24 individuals per population (with the exception of 23 Wolayta). Variants were pruned using the PLINK software [94] with parameters --indep 50 5 2 to remove the effect of linkage disequilibrium.

### SFselect and iHS

SFselect is a machine-learning site frequency spectrum-based method to detect adaptive selection in polymorphism data [48], which is available at [95]. The program was developed using supervised learning (support vector machines) trained with extensive forward population simulations. The simulations were performed under a neutral scenario and under a positive selection scenario where a selected allele experienced 200 different combinations of the parameters s (selection coefficient) and τ (time under selection). SFselect shows high power to detect positive selection compared to other tests based on the site frequency spectrum. The minimum sample size of 46 chromosomes per population provides with enough accuracy to make adaptive selection inferences based on the site frequency spectrum [96]. In this study, we used the general support vector machine trained model of SFselect and applied the test by dividing the whole genome into 30 kb windows with 5 kb overlap between windows.

We used the linkage disequilibrium-based test iHS [97] to detect recent events of selection in the five Ethiopian populations. The sample size per population is of minimum 46 chromosomes, which according to [98] provides with enough power to detect signals of positive selection with iHS (minimum of 40 chromosomes is recommended). We used the physical positions to calculate iHS since there is no specific genetic map for these populations. We used the software rehh 2.0 [99] to calculate iHS for all the variants with a minor allele frequency higher than 0.05 and excluded a variant from the calculation if a 20 kb gap was found when calculating EHHs, as they may produce biases. In addition to the iHS score per SNP, we also calculated the mean iHS score (average of iHS scores across SNPs), and the maximum iHS value and –log_10_(*p*-value) of a SNP in each 30 kb window; these windows were the same as in the SFselect analysis.

To annotate the protein-coding gene content of windows, we used bedtools 2.24.0 [100] to intersect windows with the hg19 gene annotations from RefSeq. To annotate individual variants, ANNOVAR [101] was used.

### Masking

Masking was performed as described previously [7]. African and West Asian ancestries of the Ethiopian individuals were deconvoluted using PCAdmix on 20-SNP windows. The CEU and Gumuz populations were used as surrogate sources for the West Asian component and East African component respectively. The West Asian ancestry was subsequently masked.

After the masking procedure, the proportion of West Asian ancestry in a population was estimated by averaging the proportion of masked data across each SNP. For a specific 30 kb window, the same calculation was done but only including SNPs falling in the window. Consecutive 30 kb windows under selection were merged when calculating the West Asian component proportions. A resampling analysis was used to test if the general increase of West Asian component ancestry among the significant 99.99th percentile SFselect and iHS windows was significant. We sampled the number of selected windows 10^5^ times from the genome-wide windows and calculated the mean West Asian component ancestry in each to obtain a distribution of means. The values obtained for windows were compared with this distribution.

### Simulations

To test whether demographic events could mimic the genomic patterns expected from adaptation, we performed extensive simulations using a simple demographic model that captures the key elements to define the critical values for each of the tests. We used the sequence simulator SLiM [102] to generate samples of the human neutral demography. A demographic model adapted from [49] was used, adding a simple model of admixture between a sub-Saharan population and an OoA like population [7] (as a proxy for the West Asians) 2600 years ago (Supplementary Fig. 8 in Additional File 1). For simplicity, the Amhara population was used as example to model the admixture event common to all Afroasiatic populations, using a West Asian admixture proportion of 0.54.

We next checked the validity of the model by comparing the derived site frequency spectra from the real and simulated data (Supplementary Fig. 9 in Additional File 1). The main differences between real and simulated data were seen among the singletons: a deficit of singletons was observed in the real data due to the low coverage, but otherwise the differences are very small, meaning that our model fits our data well.

There is an increase of extreme SFselect and iHS scores in our real data (Supplementary Fig. 3 in Additional File 1). The 99.99th percentile SFselect score thresholds after the neutral simulations for the Gumuz and the Afroasiatic populations are 2.24 and 2.31, respectively (Supplementary Table 1 in Additional file 2). For iHS, we calculated after the neutral simulations the 99.99th percentile of both the per SNP *p*-value distribution and the 30-kb window of the mean absolute iHS scores (for an easy comparison with SFselect). We found that for the SNP-based analysis, the 99.99th percentiles per SNP were 3.88 and 3.62 for Gumuz and the Afroasiatic populations, respectively. The window analysis set the 99.99th percentile thresholds at 2.54 and 2.53.

### Functional enrichment analysis

To understand the biological functions that may have been under positive selection, we used ClueGo [103], a Cytoscape [104] plug-in that integrates Gene Ontology, KEGG pathways Reactome and Wikipathway databases to map groups of genes to specific functions. ClueGo enables visualisation in a functionally grouped annotation network, a pie graph showing the group leading terms (most significant term among a group) and a histogram with all significant terms after p-adjustment (< 0.05 after Benjamini-Hochbert correction) and their number of genes from the analysed cluster found in our list of genes. In this case, we used the genes falling among the top 99.5 percentile of SFselect and mean iHS scores. All information about significant terms and associated genes for each population and selection tests can be found in the Additional file 3.

## Additional Files

**Additional file 1 Supplementary Fig. 1.** Location of the five sampled populations. **Supplementary Fig. 2.** Genome-wide Manhattan plots of SFselect scores of the five populations of study. **Supplementary Fig. 3.** Density plots of SFselect and iHS scores of neutral and real data. **Supplementary Fig. 4.** Principal component analysis of the five East African populations. **Supplementary Fig. 5.** ADMIXTURE analysis of the Ethiopian samples and a set of worldwide populations. **Supplementary Fig. 6.** Genome-wide Manhattan plots of the –log10(*p*-value) of iHS of the five populations of study. **Supplementary Fig. 7.** PCA of the masked East African samples with a set of Europeans and Africans. **Supplementary Fig. 8.** Schematic representation of the demographic model used to simulate neutral sequences. **Supplementary Fig. 9.** Relative site frequency spectrum of Afroasiatic and Gumuz populations**.**

**Additional file 2 Supplementary Table 1.** The 99.99*th* and 99.90*th* percentile thresholds of SFselect and iHS calculated after the neutral simulations. **Supplementary Table 2.** SFselect positive selection signals found in the five populations of study. **Supplementary Table 3.** SFselect positive selection signals found in the five populations of study. **Supplementary Table 4.** iHS positive selection signals found in the five populations of study. **Supplementary Table 5.** iHS positive selection signals shared among the five populations of study**.**

**Additional file 3 Supplementary Text**. Information about additional examples of shared and population-specific signals of positive selection.

## Data Availability

The Ethiopian datasets analysed during the current study are available in the European Genome-phenome Archive (EGA) repository with accession number EGAS00001000238.

## Abbreviations

CEU: Utah residents with Northern and Western European Ancestry
Kb: Kilobase
LD: Linkage disequilibrium
Mb: Megabase
OoA: Out of Africa
PCA: Principal Component Analysis
SNP: Single nucleotide polymorphism
UV: Ultraviolet
YRI: Yoruba from Ibadan, Nigeria
