# Supplementary material for: Positive selection in admixed populations from Ethiopia
Source: BMC Genet. 2020 Oct 22;21(Suppl 1):108. doi: 10.1186/s12863-020-00908-5 (PMC7580818; doi:10.1186/s12863-020-00908-5)
Supplement: Supplementary file 1 — Additional file 1 Supplementary Fig. 1. Location of the five sampled populations. Supplementary Fig. 2. Genome-wide Manhattan plots of SFselect scores of the five populations of study. Supplementary Fig. 3. Density plots of SFselect and iHS scores of neutral and real data. Supplementary Fig. 4. Principal component analysis of the five East African populations. Supplementary Fig. 5. ADMIXTURE analysis of the Ethiopian samples and a set of worldwide populations. Supplementary Fig. 6. Genome-wide Manhattan plots of the –log10(p-value) of iHS of the five populations of study. Supplementary Fig. 7. PCA of the masked East African samples with a set of Europeans and Africans. Supplementary Fig. 8. Schematic representation of the demographic model used to simulate neutral sequences. Supplementary Fig. 9. Relative site frequency spectrum of Afroasiatic and Gumuz populations. [file 12863_2020_908_MOESM1_ESM.pdf]

ADDITIONAL FILE 1: POSITIVE SELECTION IN ADMIXED POPULATIONS FROM ETHIOPIA

Sandra Walsh, Luca Pagani, Yali Xue, Hafid Laayouni, Chris Tyler-Smith, Jaume Bertranpetit

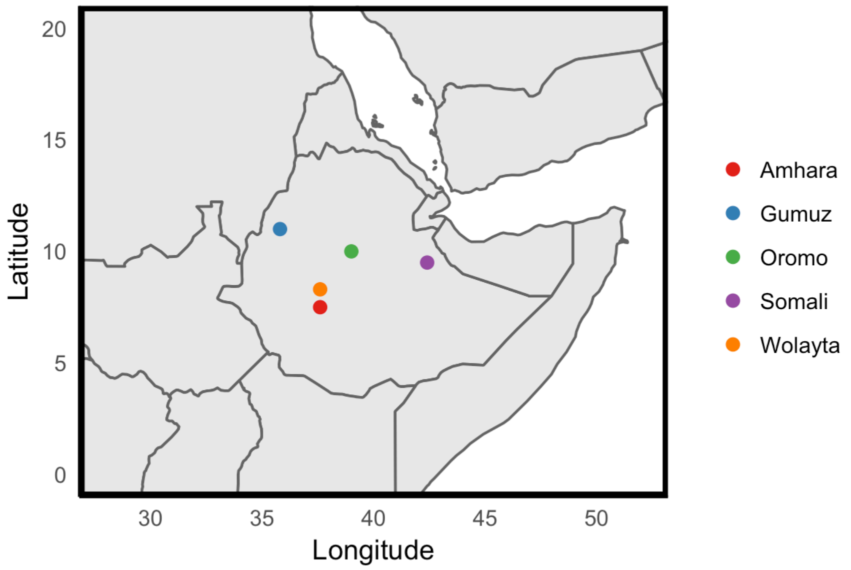

Supplementary figure 1: Location of the five sampled populations.

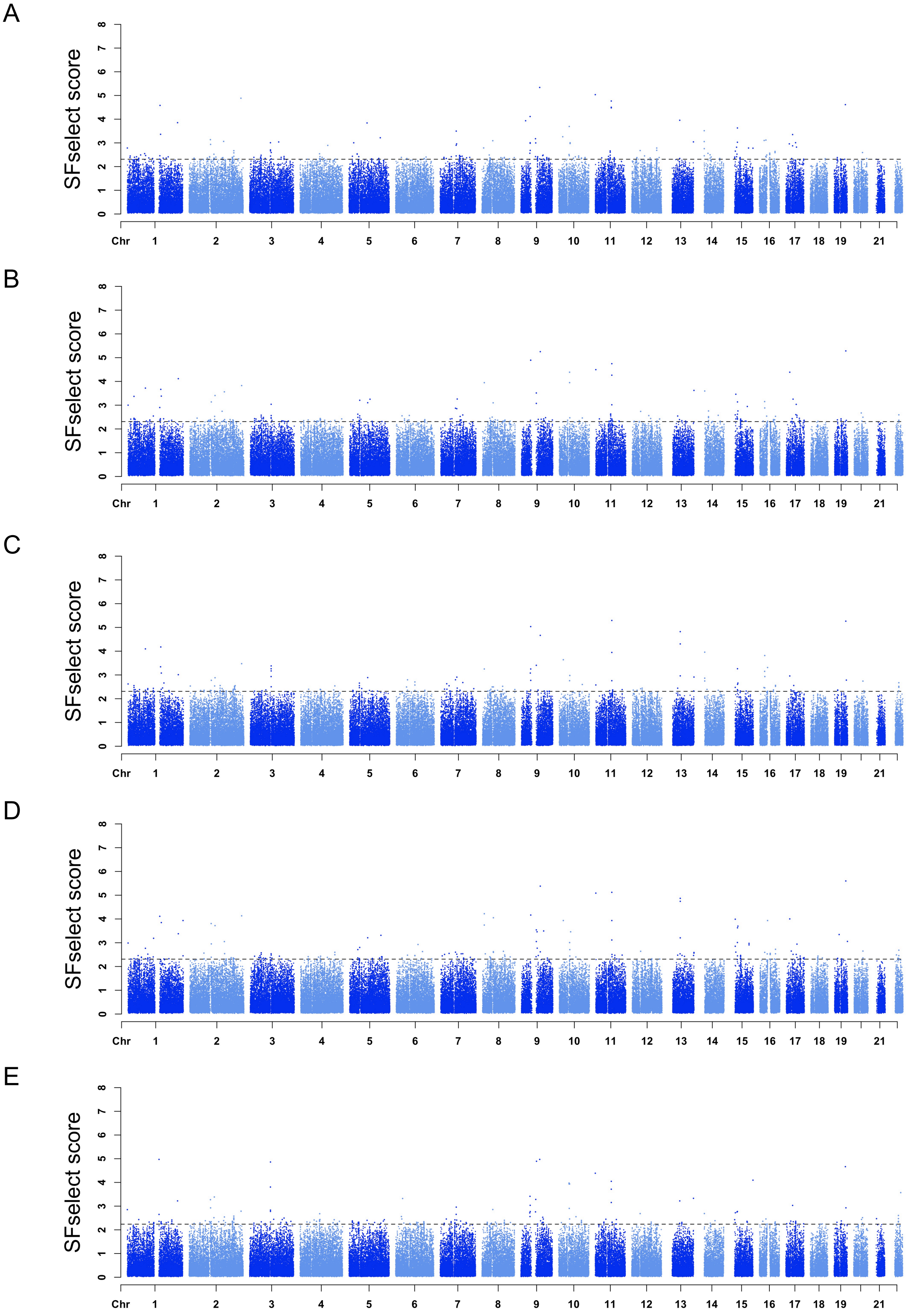

Supplementary Figure 2: Genome-wide Manhattan plots of SFselect scores A) Amhara B) Oromo C) Somali D) Wolayta E) Gumuz. Each point represents the SFselect score of a 30 kb window and the dashed black lines the 99.99th percentile threshold obtained after the neutral simulations.

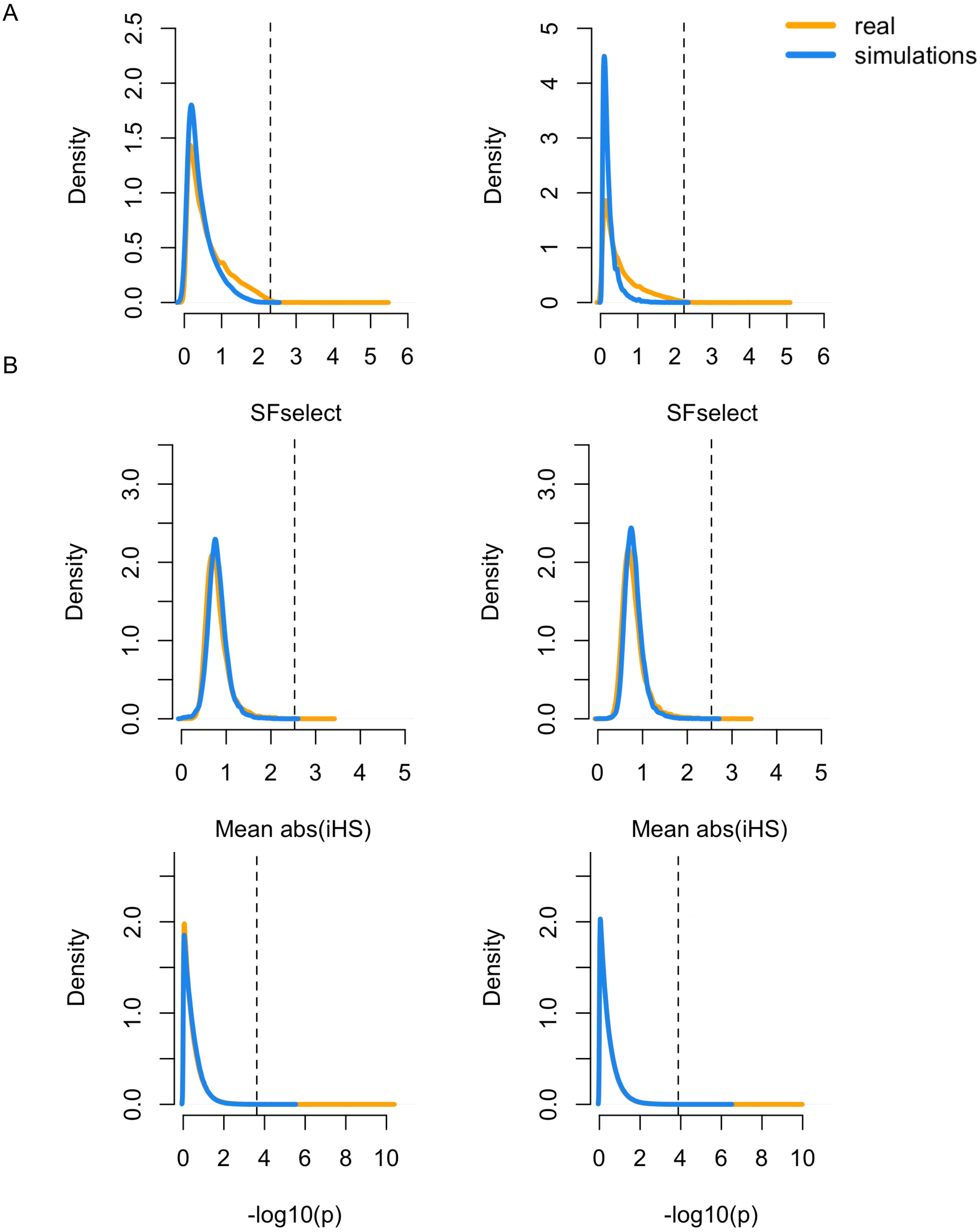

Supplementary Figure 3: Density plots of A) SFselect scores B) mean absolute iHS score in 30 kb windows C) –log10(p) per SNP, of real and simulated data for Afroasiatic (left) and Gumuz (right). The dashed line represents the 99.99th percentile threshold calculated after the simulations. Neutral and real data.

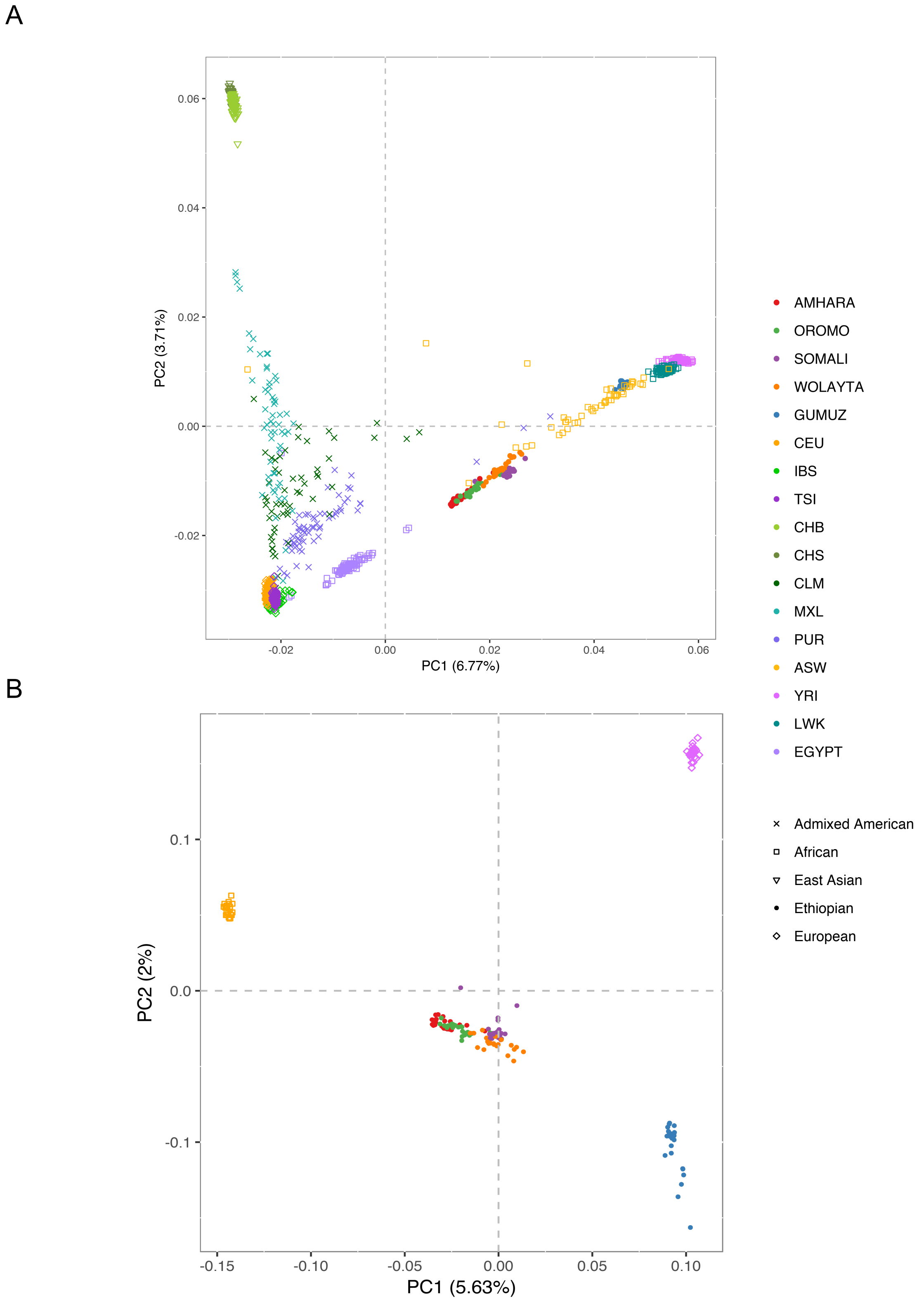

Supplementary figure 4: Principal component analysis of the five East African populations. **A**) In a worldwide context, the analysis included a set of populations from the 1000 Genomes Project. **B**) In a local context, only including the East African samples, a set of Europeans and Western Africans.

A

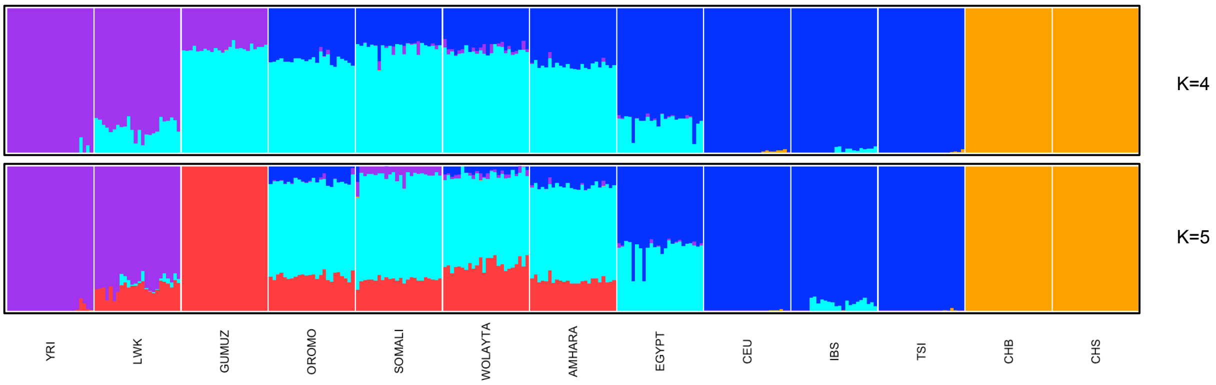

B

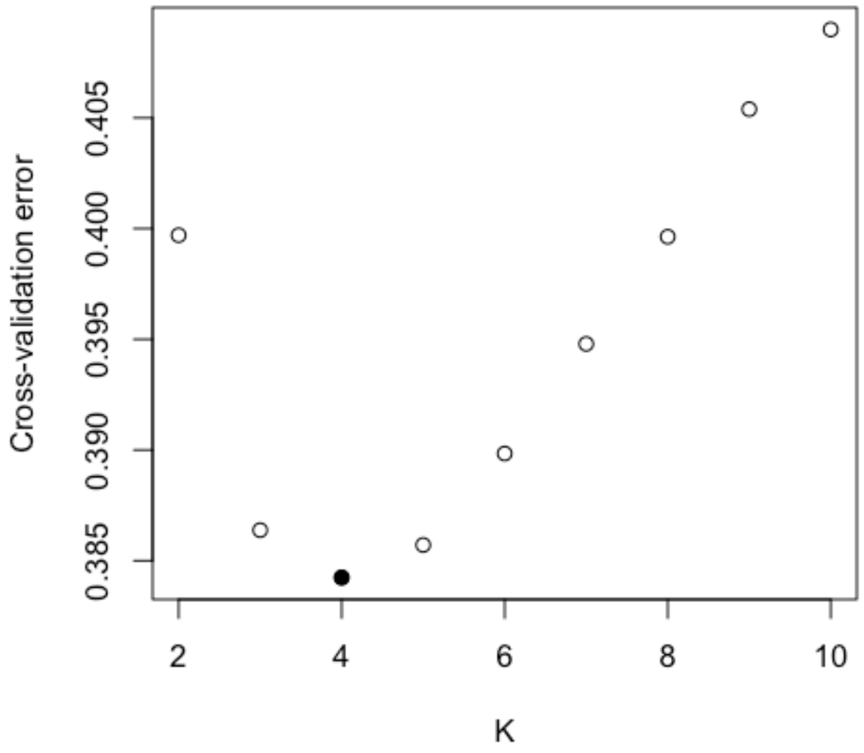

Supplementary figure 5: A) ADMIXTURE analysis of the Ethiopian samples and a set of worldwide populations. B) The lowest cross-validation error was obtained with K=4 and K=5 components. The resulting ancestry components describe Western Africa (purple), North-East Africa (light blue), East Africa (red), Europe (dark blue), East Asia (orange).

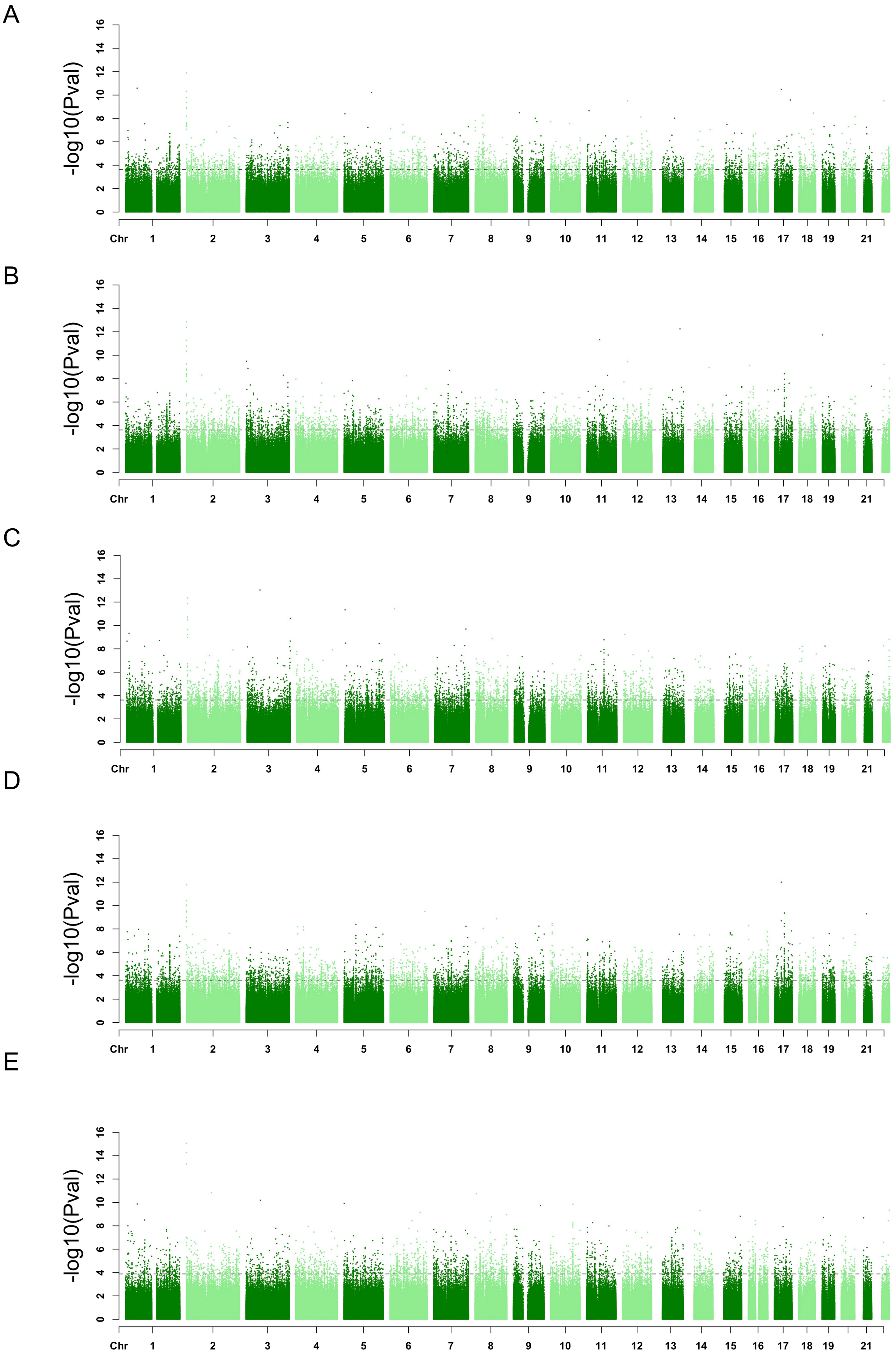

Supplementary Figure 6: Genome-wide Manhattan plots of the –log10(p-value) of iHS for A) Amhara B) Oromo C) Somali D) Wolayta E) Gumuz. Each point represents the –log10(p-value) of the iHS score of a SNP normalised by allele frequency bins of 0.05. The horizontal dashed black lines represent the 99.99[th] percentile threshold obtained after the neutral simulations.

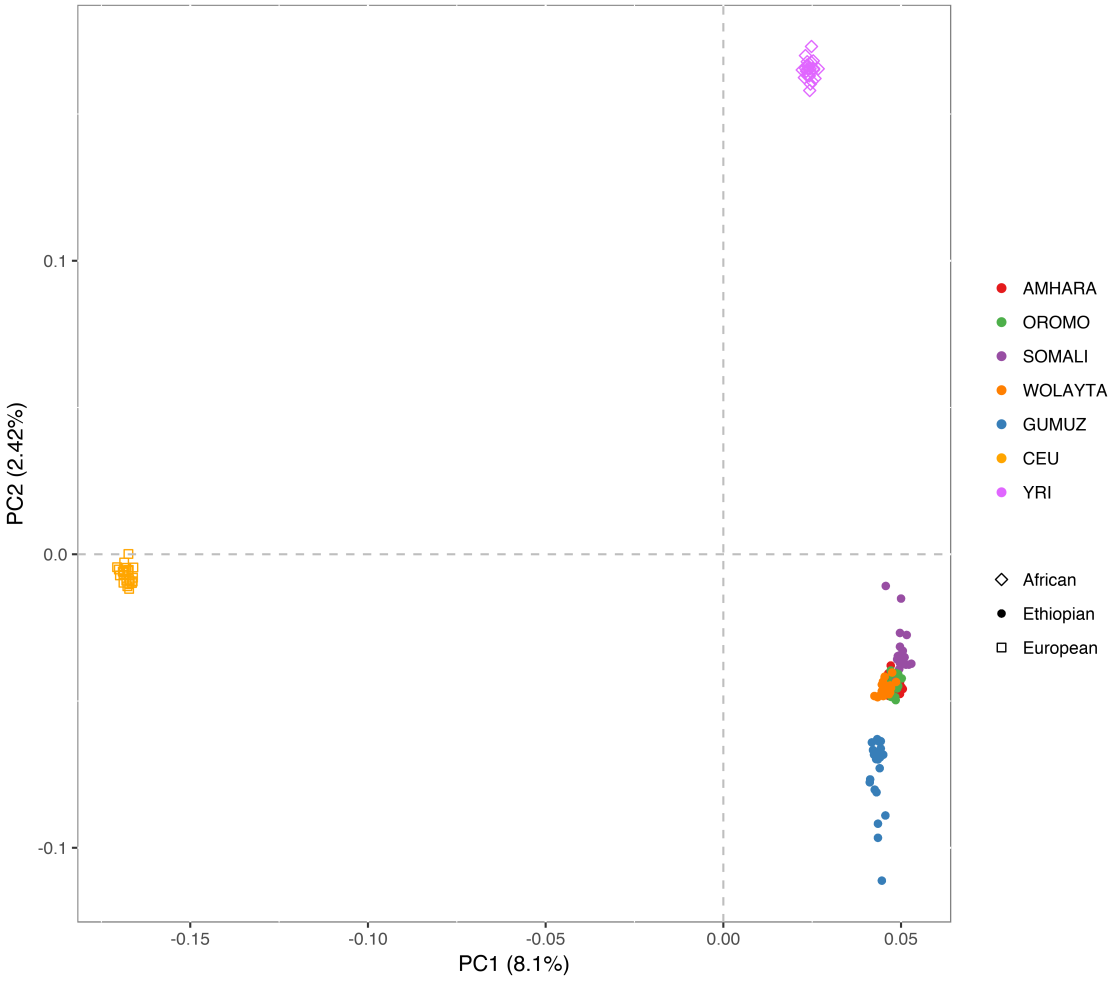

Supplementary Figure 7: PCA of the masked East African samples with a set of Europeans and Africans. The Afroasiatic samples now cluster next to the unadmixed Gumuz in comparison with Figure 2 where they clustered between Europeans and the Gumuz.

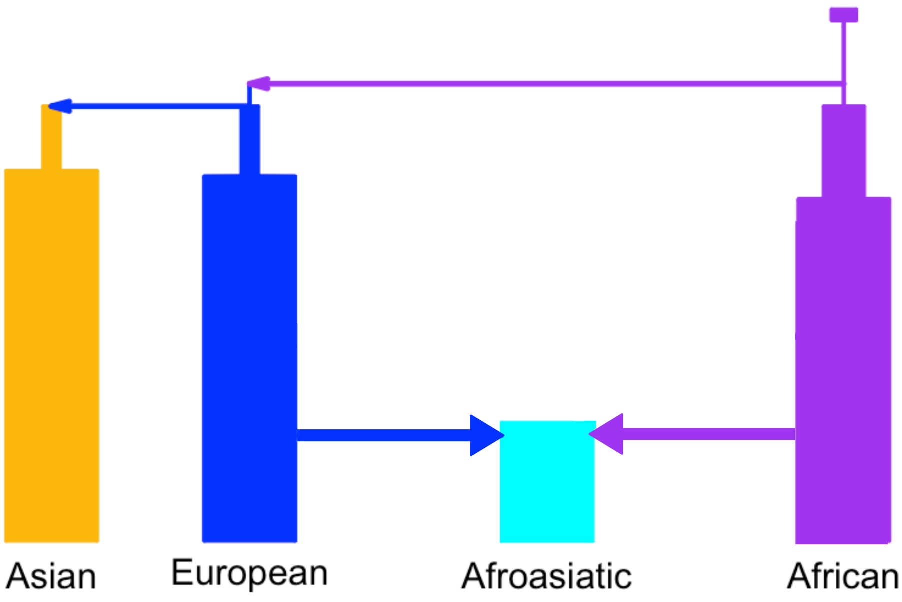

Supplementary Figure 8: Schematic representation of the demographic model used to simulate neutral sequences. Parameters where obtained from Jouganous et al 2017.

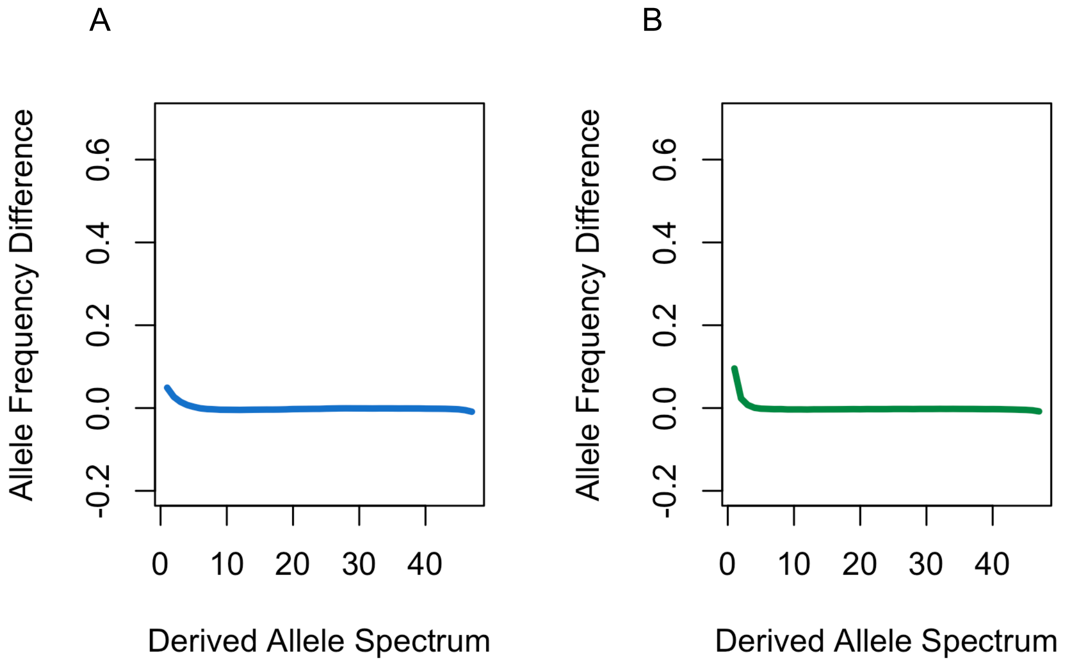

Supplementary Figure 9: Relative site frequency spectrum A) Afroasiatic B) Gumuz. The difference of derived allele frequency between neutral simulations and real data. The neutral simulations fit the real data for both Afroasiatic and Gumuz data since there are no main differences between the derived site frequency spectrum between real and simulated data. The slight increase of singletons in simulations can be explained by the lack of coverage in real data and inaccuracies of the demographic model.
