# Supplementary material for: Positive selection in admixed populations from Ethiopia
Source: BMC Genet. 2020 Oct 22;21(Suppl 1):108. doi: 10.1186/s12863-020-00908-5 (PMC7580818; doi:10.1186/s12863-020-00908-5)
Supplement: Supplementary file 2 — Additional file 2 Supplementary Table 1. The 99.99th and 99.90th percentile thresholds of SFselect and iHS calculated after the neutral simulations. Supplementary Table 2. SFselect positive selection signals found in the five populations of study. Supplementary Table 3. SFselect positive selection signals found in the five populations of study. Supplementary Table 4. iHS positive selection signals found in the five populations of study. Supplementary Table 5. iHS positive selection signals shared among the five populations of study. [file 12863_2020_908_MOESM2_ESM.pdf]

ADDITIONAL FILE 2: POSITIVE SELECTION IN ADMIXED POPULATIONS FROM ETHIOPIA

Sandra Walsh, Luca Pagani, Yali Xue, Hafid Laayouni, Chris Tyler-Smith, Jaume Bertranpetit

	SFselect		Mean iHS per window		-log10(p) iHS SNP based	
Percentile	99.99	99.90	99.99	99.90	99.99	99.90
Afroasiatic	2.31 (n= 20424)	1.93 (n= 20424)	2.53 (n=15809)	1.95 (n=15809)	3.62 (n=738269)	2.77 (n=738269)
Gumuz	2.24 (n= 24000)	1.56 (n= 24000)	2.54 (n=19240)	2.05 (n=19240)	3.88 (n=1055794)	2.98 (n= 1055794)

Supplementary Table 1: The 99.99th and 99.90th percentile thresholds of SFselect and iHS calculated after the neutral simulations. For SFselect and mean iHS we report in parenthesis the number of 30 kb simulated windows and for the SNP based iHS the number of SNPs used to calculate the thresholds.

	Chr	Start	Stop	SFselect	Genes
	chr2	232673695	232703695	4.89	*COPS7B*
	chr11	71898574	71928574	4.77	*FOLR1, FOLR2*
	chr1	147396808	147426808	4.58	*GPR89B*
	chr11	71948574	71978574	4.50	*INPPL1, PHOX2A*
	chr11	71923574	71953574	4.48	*FOLR2, INPPL1, PHOX2A*
*	chr9	21215910	21245910	3.93	*IFNA16, IFNA17, IFNA14*
	chr1	226096808	226126808	3.85	*LEFTY1, RP4-559A3.7, PYCR2, LEFTY2*
	chr5	81280629	81310629	3.84	*ATG10*
	chr15	32754330	32784330	3.63	*AC135983.2*
	chr7	72433619	72463619	3.50	*TRIM74, AC005488.1*
	chr1	149821808	149851808	3.36	*HIST2H2AA4, HIST2H3A, HIST2H4B*
	chr17	30351088	30381088	3.35	*LRRC37B*
	chr10	18164034	18194034	3.26	*MRC1*
	chr5	140705629	140735629	3.22	*PCDHGA1, PCDHGA2, PCDHGA3, PCDHGB1, PCDHGA4*
	chr8	48786376	48816376	3.09	*PRKDC*
	chr2	155023695	155053695	3.06	*GALNT13*
	chr3	130698294	130728294	3.04	*ATP2C1*
	chr17	43676088	43706088	3.01	*CRHR1*
	chr3	93598294	93628294	3.01	*PROS1*
*	chr10	51214034	51244034	3.01	*AGAP8*
	chr10	51189034	51219034	2.97	*FAM21D*
	chr17	15526088	15556088	2.96	*RP11-385D13.1, TRIM16*
	chr7	74158619	74188619	2.96	*GTF2I, NCF1*
	chr17	30326088	30356088	2.88	*SUZ12, LRRC37B*
	chr17	47876088	47906088	2.82	*KAT7*
*	chr12	112399817	112429817	2.78	*TMEM116*
	chr3	93773294	93803294	2.71	*ARL13B, DHFRL1, NSUN3*
*	chr12	112374817	112404817	2.69	*TMEM116*
	chr2	200773695	200803695	2.67	*C2orf69, TYW5*
	chr3	93798294	93828294	2.66	*NSUN3*

Supplementary Table 2A: SFselect positive selection signals found in Amhara population. All the 30 kb window scores reported are above the 99.99 percentile threshold calculated after the simulations. Only protein coding genes are reported.

* : Population-specific positive selection signals. These signals are above the 99.99 percentile threshold calculated after the simulations that are not found among the 99 percentile of the rest of the populations of study.

	Chr	Start	Stop	SFselect	Genes
	chr11	71923574	71953574	4.75	*FOLR2, INPPL1, PHOX2A*
	chr17	15526088	15556088	4.39	*RP11-385D13.1, TRIM16*
	chr10	46339034	46369034	4.39	*AGAP4*
	chr11	71948574	71978574	4.26	*INPPL1, PHOX2A*
	chr1	226096808	226126808	4.12	*LEFTY1, RP4-559A3.7, PYCR2, LEFTY2*
	chr10	46489034	46519034	3.95	*PTPN20A*
	chr2	232673695	232703695	3.83	*COPS7B*
	chr1	78321808	78351808	3.72	*FAM73A*
	chr1	147396808	147426808	3.67	*GPR89B*
	chr13	114717755	114747755	3.62	*RASA3*
	chr2	155023695	155053695	3.56	*GALNT13*
	chr2	113198695	113228695	3.41	*RGPD8*
	chr1	149796808	149826808	3.38	*HIST2H4A, HIST2H3C, HIST2H2AA3, HIST2H2AA4, HIST2H3A, HIST2H4B*
	chr1	27096808	27126808	3.37	*ARID1A, PIGV*
	chr7	74158619	74188619	3.26	*GTF2I, NCF1*
	chr17	30351088	30381088	3.25	*LRRC37B*
	chr5	45505629	45535629	3.21	*HCN1*
	chr15	30829330	30859330	3.14	*GOLGA8Q*
	chr5	81280629	81310629	3.10	*ATG10*
	chr3	93698294	93728294	3.04	*ARL13B*
	chr11	71898574	71928574	3.02	*FOLR1, FOLR2*
	chr15	74954330	74984330	2.95	*EDC3*
	chr16	22021755	22051755	2.88	*C16orf52*
	chr14	36169850	36199850	2.76	*RALGAPA1*
	chr15	32754330	32784330	2.76	*AC135983.2*
	chr2	144973695	145003695	2.74	*GTDC1*
*	chr20	32178005	32208005	2.67	*CBFA2T2*
	chr11	66898574	66928574	2.63	*KDM2A*
	chr15	30704330	30734330	2.62	*GOLGA8R*
	chr5	36880629	36910629	2.62	*NIPBL*

Supplementary Table 2B: SFselect positive selection signals found in Oromo population. All the 30 kb window scores reported are above the 99.99 percentile threshold calculated after the simulations. Only protein coding genes are reported.

* : Population-specific positive selection signals. These signals are above the 99.99 percentile threshold calculated after the simulations that are not found among the 99 percentile of the rest of the populations of study.

	Chr	Start	Stop	SFselect	Genes
	chr11	71923574	71953574	5.29	*FOLR2, INPPL1, PHOX2A*
	chr13	53167755	53197755	4.30	*HNRNPA1L2*
	chr1	147396808	147426808	4.18	*GPR89B*
	chr1	78321808	78351808	4.10	*FAM73A*
	chr11	71948574	71978574	3.95	*INPPL1, PHOX2A*
	chr16	22021755	22051755	3.82	*C16orf52*
	chr10	18164034	18194034	3.64	*MRC1*
	chr2	232673695	232703695	3.48	*COPS7B*
	chr3	93798294	93828294	3.38	*NSUN3*
	chr1	146446808	146476808	3.34	*NBPF12*
	chr15	30829330	30859330	3.26	*GOLGA8Q*
	chr3	93598294	93628294	3.26	*PROS1*
	chr3	93773294	93803294	3.17	*ARL13B, DHFRL1, NSUN3*
	chr1	149821808	149851808	3.08	*HIST2H2AA4, HIST2H3A, HIST2H4B*
	chr1	226096808	226126808	3.01	*LEFTY1, RP4-559A3.7, PYCR2, LEFTY2*
	chr17	15526088	15556088	2.96	*RP11-385D13.1, TRIM16*
*	chr16	22396755	22426755	2.93	*CDR2*
	chr13	114717755	114747755	2.91	*RASA3*
	chr7	72433619	72463619	2.91	*TRIM74, AC005488.1*
	chr5	81280629	81310629	2.89	*ATG10*
	chr2	113198695	113228695	2.88	*RGPD8*
	chr6	50655815	50685815	2.80	*TFAP2D*
	chr19	53032864	53062864	2.78	*ZNF808, ZNF701*
	chr10	46339034	46369034	2.76	*AGAP4*
	chr11	71898574	71928574	2.76	*FOLR1, FOLR2*
	chr20	39678005	39708005	2.74	*TOP1*
	chr6	83805815	83835815	2.71	*DOPEY1*
*	chr7	99258619	99288619	2.68	*CYP3A5*
	chr22	32476780	32506780	2.67	*SLC5A1*
	chr1	161546808	161576808	2.66	*FCGR3A, FCGR2B*

Supplementary Table 2C: SFselect positive selection signals found in Somali population. All the 30 kb window scores reported are above the 99.99 percentile threshold calculated after the simulations. Only protein coding genes are reported.

* : Population-specific positive selection signals. These signals are above the 99.99 percentile threshold calculated after the simulations that are not found among the 99 percentile of the rest of the populations of study.

	Chr	Start	Stop	SFselect	Genes
	chr11	71923574	71953574	5.12	*FOLR2, INPPL1, PHOX2A*
	chr13	53167755	53197755	4.74	*HNRNPA1L2*
	chr2	232673695	232703695	4.13	*COPS7B*
	chr8	48786376	48816376	4.05	*PRKDC*
	chr17	15526088	15556088	4.00	*RP11-385D13.1, TRIM16*
	chr10	18164034	18194034	3.93	*MRC1*
	chr11	71948574	71978574	3.93	*INPPL1, PHOX2A*
	chr1	149796808	149826808	3.85	*HIST2H4A, HIST2H3C, HIST2H2AA3, HIST2H2AA4, HIST2H3A, HIST2H4B*
	chr2	95748695	95778695	3.81	*MRPS5*
	chr2	113198695	113228695	3.72	*RGPD8*
	chr15	32754330	32784330	3.70	*AC135983.2*
	chr15	30829330	30859330	3.63	*GOLGA8Q*
	chr9	99740910	99770910	3.50	*HIATL2*
	chr10	51189034	51219034	3.46	*FAM21D*
	chr1	226096808	226126808	3.38	*LEFTY1, RP4-559A3.7, PYCR2, LEFTY2*
	chr5	140705629	140735629	3.31	*PCDHGA1, PCDHGA2, PCDHGA3, PCDHGB1, PCDHGA4*
	chr5	81280629	81310629	3.21	*ATG10*
*	chr1	115121808	115151808	3.19	*BCAS2, DENND2C*
	chr11	71898574	71928574	3.12	*FOLR1, FOLR2*
*	chr19	57907864	57937864	3.06	*AC003002.4, ZNF548, AC003002.6, AC004076.7, ZNF17*
	chr2	155023695	155053695	3.05	*GALNT13*
	chr10	46489034	46519034	3.01	*PTPN20A*
	chr17	47876088	47906088	2.94	*KAT7*
	chr5	45355629	45385629	2.80	*HCN1*
	chr1	78321808	78351808	2.77	*FAM73A*
	chr16	70346755	70376755	2.73	*DDX19B, RP11-529K1.3*
	chr5	36955629	36985629	2.71	*NIPBL*
	chr22	32476780	32506780	2.69	*SLC5A1*
	chr20	39678005	39708005	2.65	*TOP1*
	chr6	118030815	118060815	2.63	*NUS1*

Supplementary Table 2D: SFselect positive selection signals found in Wolayta population. All the 30 kb window scores reported are above the 99.99 percentile threshold calculated after the simulations. Only protein coding genes are reported.

* : Population-specific positive selection signals. These signals are above the 99.99 percentile threshold calculated after the simulations that are not found among the 99 percentile of the rest of the populations of study.

	Chr	Start	Stop	SFselect	Genes
	chr3	93798294	93828294	4.86	*NSUN3*
	chr11	71948574	71978574	4.05	*INPPL1, PHOX2A*
	chr10	46489034	46519034	3.97	*PTPN20A*
	chr10	46539034	46569034	3.93	*PTPN20A*
	chr3	93598294	93628294	3.81	*PROS1*
	chr11	71898574	71928574	3.71	*FOLR1, FOLR2*
*	chr9	40790910	40820910	3.41	*ZNF658*
	chr2	113198695	113228695	3.38	*RGPD8*
	chr13	114717755	114747755	3.33	*RASA3*
	chr2	95748695	95778695	3.27	*MRPS5*
	chr1	226096808	226126808	3.22	*LEFTY1, RP4-559A3.7, PYCR2, LEFTY2*
	chr11	71923574	71953574	3.15	*FOLR2, INPPL1, PHOX2A*
	chr17	30351088	30381088	3.03	*LRRC37B*
	chr7	72433619	72463619	2.96	*TRIM74, AC005488.1*
	chr19	53032864	53062864	2.93	*ZNF808, ZNF701*
	chr8	48786376	48816376	2.86	*PRKDC*
*	chr3	93723294	93753294	2.83	*ARL13B, STX19*
*	chr2	233223695	233253695	2.79	*ALPP*
	chr3	93748294	93778294	2.78	*ARL13B, DHFRL1*
	chr15	32754330	32784330	2.77	*AC135983.2*
	chr15	30704330	30734330	2.75	*GOLGA8R*
	chr4	88110998	88140998	2.68	*KLHL8*
	chr22	32451780	32481780	2.61	*SLC5A1*
	chr2	200798695	200828695	2.58	*C2orf69, TYW5, C2orf47*
*	chr9	40865910	40895910	2.56	*ZNF658*
	chr6	25955815	25985815	2.56	*TRIM38*
	chr10	77464034	77494034	2.55	*C10orf11*
*	chr2	158448695	158478695	2.54	*ACVR1C*
	chr9	97990910	98020910	2.53	*FANCC*
	chr2	111373695	111403695	2.51	*BUB1*

Supplementary Table 2E: SFselect positive selection signals found in Gumuz population. All the 30 kb window scores reported are above the 99.99 percentile threshold calculated after the simulations. Only protein coding genes are reported.

* : Population-specific positive selection signals. These signals are above the 99.99 percentile threshold calculated after the simulations that are not found among the 99 percentile of the rest of the populations of study.

Chr	Start	End	Amhara	Oromo	Somali	Wolayta	Gumuz	Genes	Type of gene
chr9	84515910	84545910	5.34	5.25	4.67	5.38	4.97	*RP11-383M4.2, RP11-383M4.6, SPATA31D4, SPATA31D5P*	lincRNA, lincRNA, PS,PS
chr11	123574	153574	5.04	4.50	2.58	5.09	4.39	*LINC01001, RP11-304M2.6, CICP23, RNU6-447P*	lincRNA, lincRNA, PS, snRNA
chr11	71898574	71928574	4.77	3.02	2.76	3.12	3.71	*FOLR1, FOLR2*	PC
chr19	50582864	50612864	4.61	4.67	5.28	5.26	5.60	-	-
chr11	71948574	71978574	4.50	4.26	3.95	3.93	4.05	*INPPL1, PHOX2A, AP000593.5*	PC, PC, PS
chr11	71923574	71953574	4.48	4.75	5.29	5.12	3.15	*FOLR2, INPPL1, PHOX2A*	PC
chr1	226096808	226126808	3.85	4.12	3.01	3.38	3.22	*LEFTY1, LEFTY2, PYCR2*	PC
chr5	81280629	81310629	3.84	3.10	2.89	3.21	2.40	*ATG10, PPIAP11*	PC, PS
chr15	32754330	32784330	3.63	2.76	2.66	3.70	2.77	*AC135983.2, RP11-632K20.6*	PC, PS
chr3	93598294	93628294	3.01	2.47	3.26	2.54	3.81	*PROS1*	PC
chr2	96448695	96478695	2.94	3.14	2.77	2.95	2.93	*LINC00342, AC008268.2*	lincRNA, PS
chr1	571808	601808	2.78	3.00	2.62	2.99	2.85	*RP5-857K21.4*	lincRNA
chr20	39678005	39708005	2.59	2.49	2.74	2.65	2.51	*TOP1*	PC
chr15	30704330	30734330	2.52	2.62	2.38	2.45	2.75	*GOLGA8R, RP11-382B18.5*	PC, PS
chr4	89360998	89390998	2.42	2.40	2.38	2.43	2.44	*HERC5, HERC6*	PC
chr1	184021808	184051808	2.41	2.32	2.34	2.33	2.32	*TSEN15*	PC

Supplementary Table 3: SFselect positive selection signals shared among the five populations of study. All the reported SFselect scores of the 30 kb windows are above the 99.99 percentile threshold calculated after the simulations. We also report the genes within the window and the type of gene (PC: protein coding gene, PS: pseudogene, lincRNA: long interspersed non-coding RNA, small nuclear RNA: snRNA).

			Amhara		Oromo		Somali		Wolayta		Gumuz			
Chr	Start	End	mean	maxP	mean	maxP	mean	maxP	mean	maxP	mean	maxP	Genes	Type of gene
chr16	21746755	21776755	3.12	6.59	2.85	7.32	2.82	5.50	2.32	6.02	3.20	7.05	*OTOA*	PC
chr8	36136376	36166376	3.09	8.28	2.88	5.62	2.42	5.16	3.17	6.46	2.29	3.98	*MTND6P19, RP11-139F9.1, RP11-593P24.2, RP11-593P24.3*	PS
chr16	32296755	32326755	2.97	6.71	2.98	5.29	3.52	6.31	3.05	5.61	3.56	8.45	*RP11-17M15.2*	PS
chr16	33496755	33526755	2.54	4.87	2.53	5.34	2.88	5.11	2.46	4.70	2.06	5.21	*BMS1P8*	PS
chr7	65258619	65288619	2.45	5.09	2.66	7.49	2.13	5.39	2.66	6.28	2.74	5.27	*RNU6-912P, RP11-479O9.2*	snRNA, PS
chr16	32121755	32151755	2.35	2.88	2.53	5.37	2.08	3.06	2.62	3.38	2.89	3.67	*HERC2P4, RP11-1166P10.7*	PS
chr16	32321755	32351755	2.29	6.71	2.06	5.29	2.29	6.12	2.19	5.61	2.05	5.61	*RP11-17M15.2*	PS
chr4	510998	540998	2.24	5.67	2.30	3.71	2.68	6.25	2.12	3.54	2.35	4.53	*PIGG*	PC
chr2	398695	428695	2.14	11.89	2.95	12.85	2.55	12.36	2.36	11.78	2.45	15.05	*AC105393.1, AC105393.2*	lincRNA
chr16	31996755	32026755	1.95	2.23	2.61	2.59	2.25	3.08	2.37	3.01	3.37	4.47	*RP11-1166P10.1, RP11-1166P10.6*	PS

Supplementary Table 4: iHS positive selection signals shared among the five populations of study. For each population the mean iHS score for the 30 kb windows is reported and the highest –log(p-value) of a variant within the window. The windows were selected for comparison if their mean iHS score was above the 99.99 percentile threshold in at least one population and in the 99.9 threshold in the rest. In gray, the only shared window that is in all populations with mean iHS scores above the 99.99 threshold. We also report the genes within the window and the type of gene (PC: protein coding gene, PS: pseudogene, lincRNA: long interspersed non-coding RNA, small nuclear RNA: snRNA).

	Chr	Start	Stop	meaniHS	maxP	Genes
	chr18	6247301	6277301	4.14	4.67	*L3MBTL4*
	chr1	202396808	202426808	3.57	6.71	*PPP1R12B*
	chr18	66647301	66677301	3.34	6.67	*CCDC102B*
	chr16	21746755	21776755	3.12	6.58	*OTOA*
	chr9	74490910	74520910	3.05	4.08	*ABHD17B*
	chr1	244721808	244751808	2.99	5.66	*C1orf101*
	chr18	66722301	66752301	2.98	8.46	*CCDC102B*
	chr1	244696808	244726808	2.96	5.73	*C1orf101*
	chr6	128505815	128535815	2.92	4.82	*PTPRK*
	chr11	85173574	85203574	2.92	5.16	*DLG2*
	chr12	22524817	22554817	2.89	9.50	*ST8SIA1*
	chr12	113674817	113704817	2.86	5.59	*TPCN1*
	chr1	244671808	244701808	2.77	5.18	*C1orf101*
	chr6	157480815	157510815	2.73	4.75	*ARID1B*
*	chr6	136905815	136935815	2.71	6.33	*MAP3K5*
	chr2	158323695	158353695	2.71	4.83	*CYTIP*
	chr12	113699817	113729817	2.68	4.06	*TPCN1*
	chr11	75573574	75603574	2.66	4.31	*UVRAG*
*	chr4	47210998	47240998	2.63	4.79	*GABRB1*
	chr22	46701780	46731780	2.58	4.62	*GTSE1, TRMU*
	chr9	40815910	40845910	2.54	5.23	*ZNF658*
	chr11	85198574	85228574	2.53	4.76	*DLG2*

Supplementary Table 5A: iHS positive selection signals found in Amhara population. The mean iHS score for the 30 kb windows is reported and the highest –log(p-value) of a variant within the window. All the reported iHS scores are above the reported 99.99 percentile thresholds calculated after the simulations. Only protein coding genes are reported.

* : Population-specific positive selection signals. These signals are above the 99.99 percentile threshold calculated after the simulations that are not found among the 99 percentile of the rest of the populations of study.

	Chr	Start	Stop	meaniHS	maxP	Genes
	chr3	19248294	19278294	3.33	7.46	*KCNH8*
	chr1	202396808	202426808	3.14	6.77	*PPP1R12B*
	chr17	79776088	79806088	3.08	4.25	*FAM195B, AC174470.1, PPP1R27, P4HB*
	chr2	237448695	237478695	3.07	5.37	*ACKR3*
	chr12	8299817	8329817	2.96	2.77	*ZNF705A*
	chr11	72023574	72053574	2.94	6.60	*CLPB*
	chr3	19298294	19328294	2.91	4.79	*KCNH8*
	chr2	158323695	158353695	2.87	6.16	*CYTIP*
	chr11	72048574	72078574	2.86	4.86	*CLPB*
	chr16	21746755	21776755	2.85	7.32	*OTOA*
	chr12	82849817	82879817	2.82	7.11	*METTL25*
	chr17	44351088	44381088	2.72	8.42	*ARL17B, LRRC37A*
	chr3	19273294	19303294	2.69	4.79	*KCNH8*
	chr11	75473574	75503574	2.60	3.09	*DGAT2*
	chr11	75523574	75553574	2.60	7.06	*UVRAG*
	chr3	19173294	19203294	2.59	5.31	*KCNH8*
	chr12	45674817	45704817	2.58	3.82	*ANO6*
	chr12	113674817	113704817	2.56	3.62	*TPCN1*
	chr1	149821808	149851808	2.55	3.95	*HIST2H2AA4, HIST2H3A, HIST2H4B*
	chr12	64224817	64254817	2.55	5.39	*SRGAP1*
	chr10	70414034	70444034	2.54	4.44	*TET1*
*	chr3	164673294	164703294	2.54	4.79	*SI*
	chr15	44879330	44909330	2.54	4.07	*SPG11*
	chr12	82799817	82829817	2.54	5.21	*METTL25*

Supplementary Table 5B: iHS positive selection signals found in Oromo population. The mean iHS score for the 30 kb windows is reported and the highest –log(p-value) of a variant within the window. All the reported iHS scores are above the reported 99.99 percentile thresholds calculated after the simulations. Only protein coding genes are reported.

* : Population-specific positive selection signals. These signals are above the 99.99 percentile threshold calculated after the simulations that are not found among the 99 percentile of the rest of the populations of study.

	Chr	Start	Stop	meaniHS	maxP	Genes
	chr11	75448574	75478574	3.52	7.94	*DGAT2*
	chr11	75523574	75553574	3.40	8.77	*UVRAG*
	chr2	136248695	136278695	3.32	5.99	*ZRANB3*
	chr11	75498574	75528574	3.22	6.24	*DGAT2, UVRAG*
	chr21	30808956	30838956	3.17	5.72	*BACH1*
	chr1	51596808	51626808	3.13	3.72	*C1orf185*
	chr1	70471808	70501808	3.12	6.91	*LRRC7*
	chr2	135973695	136003695	3.10	5.40	*ZRANB3*
	chr11	75548574	75578574	3.04	7.65	*UVRAG*
*	chr2	136273695	136303695	2.98	6.50	*ZRANB3, R3HDM1*
	chr11	75723574	75753574	2.96	3.80	*UVRAG*
	chr2	136073695	136103695	2.94	5.07	*ZRANB3*
*	chr9	28615910	28645910	2.94	5.38	*LINGO2*
*	chr9	28640910	28670910	2.94	5.08	*LINGO2*
	chr2	237448695	237478695	2.92	4.86	*ACKR3*
	chr2	136023695	136053695	2.91	4.86	*ZRANB3*
*	chr19	3232864	3262864	2.88	6.38	*CELF5*
*	chr15	42654330	42684330	2.86	5.87	*CAPN3*
	chr12	44674817	44704817	2.85	6.04	*TMEM117*
	chr9	40815910	40845910	2.85	5.65	*ZNF658*
	chr11	75473574	75503574	2.83	3.54	*DGAT2*
	chr1	51696808	51726808	2.83	4.72	*RNF11*
	chr16	21746755	21776755	2.82	5.50	*OTOA*
	chr12	56724817	56754817	2.82	3.85	*PAN2, IL23A, STAT2, APOF*
	chr2	136123695	136153695	2.82	5.74	*ZRANB3*
	chr4	71635998	71665998	2.80	5.71	*RUFY3*
	chr17	58826088	58856088	2.75	4.69	*BCAS3*
	chr4	71610998	71640998	2.75	6.67	*RUFY3*
	chr2	135998695	136028695	2.73	5.40	*ZRANB3*
*	chr10	57364034	57394034	2.72	2.85	*PCDH15*
	chr2	136098695	136128695	2.72	4.33	*ZRANB3*
	chr7	137258619	137288619	2.71	6.41	*DGKI*
	chr4	510998	540998	2.68	6.25	*PIGG*
	chr9	74490910	74520910	2.68	3.93	*ABHD17B*
*	chr2	135823695	135853695	2.67	3.93	*RAB3GAP1*
	chr9	74515910	74545910	2.66	4.85	*ABHD17B, C9orf85*
	chr12	45799817	45829817	2.66	5.86	*ANO6*
	chr1	51571808	51601808	2.65	4.53	*C1orf185*
*	chr13	52942755	52972755	2.63	2.27	*THSD1*
	chr7	123658619	123688619	2.63	7.45	*TMEM229A*
	chr8	145211376	145241376	2.62	5.91	*MROH1*
*	chr1	225646808	225676808	2.61	4.72	*ENAH*
	chr2	136148695	136178695	2.59	4.94	*ZRANB3*
*	chr2	135773695	135803695	2.57	4.89	*MAP3K19*
	chr12	11174817	11204817	2.57	5.91	*PRR4, TAS2R14, TAS2R19, TAS2R31, AC018630.1*
	chr4	159235998	159265998	2.56	5.33	*RXFP1*
	chr8	145161376	145191376	2.55	7.43	*SHARPIN, MAF1, KIAA1875*
	chr11	75573574	75603574	2.54	5.48	*UVRAG*
	chr7	137233619	137263619	2.53	6.41	*DGKI*
	chr7	74558619	74588619	2.53	3.73	*GTF2IRD2B*

Supplementary Table 5C: iHS positive selection signals found in Somali population. The mean iHS score for the 30 kb windows is reported and the highest –log(p-value) of a variant within the window. All the reported iHS scores are above the reported 99.99 percentile thresholds calculated after the simulations. Only protein coding genes are reported.

* : Population-specific positive selection signals. These signals are above the 99.99 percentile threshold calculated after the simulations that are not found among the 99 percentile of the rest of the populations of study.

	Chr	Start	Stop	meaniHS	maxP	Genes
	chr1	202396808	202426808	3.59	6.66	*PPP1R12B*
	chr18	66647301	66677301	3.41	6.40	*CCDC102B*
	chr17	44351088	44381088	3.20	9.36	*ARL17B, LRRC37A*
	chr19	39757864	39787864	3.14	5.36	*IFNL2, IFNL1*
	chr18	66722301	66752301	3.09	7.24	*CCDC102B*
	chr1	149821808	149851808	3.03	5.01	*HIST2H2AA4, HIST2H3A, HIST2H4B*
	chr12	82849817	82879817	2.98	6.06	*METTL25*
	chr17	30276088	30306088	2.82	4.76	*SUZ12*
*	chr11	71823574	71853574	2.82	2.76	*ANAPC15, FOLR3*
	chr17	63001088	63031088	2.82	6.13	*GNA13*
	chr8	145211376	145241376	2.81	6.46	*MROH1*
	chr17	44326088	44356088	2.81	9.36	*ARL17B*
	chr9	16565910	16595910	2.79	6.40	*BNC2*
	chr5	144980629	145010629	2.74	5.92	*PRELID2*
	chr8	124261376	124291376	2.73	5.69	*ZHX1-C8ORF76, ZHX1*
	chr18	66672301	66702301	2.72	5.42	*CCDC102B*
	chr17	62501088	62531088	2.72	2.92	*DDX5, CEP95*
	chr12	22824817	22854817	2.69	4.37	*ETNK1*
	chr8	124211376	124241376	2.68	4.99	*FAM83A, C8orf76, ZHX1-C8ORF76*
	chr2	173473695	173503695	2.65	4.94	*PDK1*
	chr1	51571808	51601808	2.63	4.00	*C1orf185*
	chr20	20678005	20708005	2.62	4.46	*RALGAPA2*
	chr20	20653005	20683005	2.62	3.97	*RALGAPA2*
	chr17	74751088	74781088	2.60	4.32	*MFSD11*
	chr8	124236376	124266376	2.60	4.61	*C8orf76, ZHX1-C8ORF76, ZHX1*
*	chr16	85621755	85651755	2.58	7.75	*GSE1*
*	chr10	45789034	45819034	2.58	3.33	*OR13A1*
	chr1	51696808	51726808	2.57	4.10	*RNF11*
	chr3	19298294	19328294	2.56	5.36	*KCNH8*
	chr12	86999817	87029817	2.56	6.20	*MGAT4C*
	chr3	19248294	19278294	2.54	4.84	*KCNH8*

Supplementary Table 5D: iHS positive selection signals found in Wolayta population. The mean iHS score for the 30 kb windows is reported and the highest –log(p-value) of a variant within the window. All the reported iHS scores are above the reported 99.99 percentile thresholds calculated after the simulations. Only protein coding genes are reported.

* : Population-specific positive selection signals. These signals are above the 99.99 percentile threshold calculated after the simulations that are not found among the 99 percentile of the rest of the populations of study.

	Chr	Start	Stop	meaniHS	maxP	Genes
*	chr10	102014034	102044034	3.74	5.78	*CWF19L1, BLOC1S2*
*	chr10	101939034	101969034	3.45	5.57	*ERLIN1, CHUK*
*	chr10	101964034	101994034	3.37	9.85	*CHUK, CWF19L1*
	chr1	202396808	202426808	3.33	5.98	*PPP1R12B*
	chr20	20528005	20558005	3.31	7.06	*RALGAPA2*
	chr16	21746755	21776755	3.20	7.05	*OTOA*
*	chr10	101989034	102019034	3.16	6.87	*CHUK, CWF19L1*
*	chr6	128080815	128110815	3.14	6.05	*THEMIS*
*	chr10	101914034	101944034	3.11	6.23	*ERLIN1*
*	chr7	12258619	12288619	3.05	7.44	*TMEM106B*
	chr8	145161376	145191376	3.05	8.95	*SHARPIN, MAF1, KIAA1875*
	chr12	113849817	113879817	3.05	5.31	*SDS, SDSL*
*	chr6	128005815	128035815	3.00	5.00	*THEMIS*
	chr12	113824817	113854817	2.92	7.44	*PLBD2, SDS*
*	chr4	130010998	130040998	2.89	5.75	*SCLT1, C4orf33*
	chr9	16565910	16595910	2.86	7.70	*BNC2*
*	chr6	154330815	154360815	2.79	5.43	*OPRM1*
	chr2	229773695	229803695	2.75	6.00	*PID1*
*	chr16	23096755	23126755	2.75	5.04	*USP31*
*	chr16	23071755	23101755	2.73	5.11	*USP31*
	chr7	120983619	121013619	2.72	3.66	*FAM3C*
	chr6	128105815	128135815	2.70	6.05	*THEMIS*
	chr16	32046755	32076755	2.69	3.77	*AC142381.1*
	chr2	98848695	98878695	2.69	4.35	*VWA3B*
*	chr6	128055815	128085815	2.68	4.27	*THEMIS*
	chr7	74558619	74588619	2.68	3.41	*GTF2IRD2B*
	chr8	145236376	145266376	2.67	4.31	*MROH1*
*	chr7	65883619	65913619	2.67	4.09	*TPST1*
*	chr6	154305815	154335815	2.67	5.17	*OPRM1*
*	chr16	23121755	23151755	2.64	5.03	*USP31*
	chr2	98823695	98853695	2.63	4.54	*VWA3B*
*	chr7	133983619	134013619	2.62	5.29	*SLC35B4*
	chr11	16048574	16078574	2.61	5.27	*SOX6*
*	chr10	102039034	102069034	2.58	8.06	*BLOC1S2, PKD2L1*
*	chr6	33030815	33060815	2.56	6.90	HLA-DPA1, HLA-DPB1
	chr7	121008619	121038619	2.56	3.66	FAM3C
*	chr4	41985998	42015998	2.55	4.93	DCAF4L1, SLC30A9

Supplementary Table 5E: iHS positive selection signals found in Gumuz population. The mean iHS score for the 30 kb windows is reported and the highest –log(p-value) of a variant within the window. All the reported iHS scores are above the reported 99.99 percentile thresholds calculated after the simulations. Only protein coding genes are reported.
* : Population-specific positive selection signals. These signals are above the 99.99 percentile threshold calculated after the simulations that are not found among the 99 percentile of the rest of the populations of study.
