# Supplementary material for: Positive selection in admixed populations from Ethiopia
Source: BMC Genet. 2020 Oct 22;21(Suppl 1):108. doi: 10.1186/s12863-020-00908-5 (PMC7580818; doi:10.1186/s12863-020-00908-5)
Supplement: Supplementary file 3 — Additional file 3 Supplementary Text. Information about additional examples of shared and population-specific signals of positive selection. [file 12863_2020_908_MOESM3_ESM.pdf]

SUPPLEMENTARY TEXT

POSITIVE SELECTION IN ADMIXED POPULATIONS FROM ETHIOPIA

Sandra Walsh, Luca Pagani, Yali Xue, Hafid Laayouni, Chris Tyler-Smith, Jaume Bertranpetit

Structure of the Ethiopian populations

Principal Component Analysis (PCA) of the five Ethiopian populations, together with 1000 Genomes Project samples (Supplementaty Figure 2a), shows PC1 (6.7% of variation) separating the African from OoA populations, and PC2 (3.6% of variation) distinguishing among the non-Africans. In PC1, the Northern Africans (Egypt) lie on the left, close to Europeans, and the sub-Saharan Yoruba on the far right. Ethiopians are widely spread between these extremes, illustrating their high genetic variation and complexity mostly due to variable amounts of Eurasian admixture [1]. Interestingly, most of the Ethiopian populations (all the Afroasiatic speakers, but not the Gumuz) lie the closest to North Africans (and other non-Africans) of all the African groups, compatible with their extensive recent OoA admixture. Although the Afroasiatic samples cluster together, the Gumuz population lies closer to the Luhya and Yoruba than to any other samples, showing again the correlation between genetic and linguistic stratification in Ethiopia.

In order to further understand the Ethiopian samples, we performed a PCA with only these five populations, the Yoruba and a European population (CEU). Supplementary Figure 2b shows that the first component (5.6% of the variation) differentiates European from African populations, and thus between Afroasiatic and Nilotic populations, revealing that

linguistic affiliation correlates with genetic similarity in Ethiopia. PC2 (2% of the variation) separates Gumuz from Yoruba, with the Afroasiatic populations in between. Among the Ethiopian Afroasiatic samples, Oromo and Amhara appear to be the most closely related with a strong overlap in the PCA.

In an ADMIXTURE analysis of a reduced set of thirteen populations (the Ethiopian samples, Egyptians and some from the 1000 Genomes Project), the clustering obtained with the best K value (K=4) showed two similar main components in the Afroasiatic samples, an Ethiopian component (light blue) at 60-70% and a second component (dark blue) shared among Europeans and North Africans. Interestingly, the Nilotic samples (Gumuz) share the same 60-70% Ethiopian component but the remaining component is shared with the Niger-Kordofanian-speaking groups Yoruba and Luhya, indicating two different genetic components in sub-Saharan Africans. Almost as significant as K=4 (Supplementary Figure 3b), K=5 shows additional features. The Gumuz samples now have a single component (red) whereas the Afroasiatic populations show in addition a component similar to Europeans (dark blue), and a North African component (light blue).

SFselect selection analysis – additional examples

We found several windows under positive selection shared between some of the populations. One example is a window containing *NSUN3* and *DHFRL1* that is shared between the Amhara, Oromo, Somali and Gumuz populations with significant scores except for Oromo that falls close to the 99.99 percentile. It is the highest scoring protein coding signal in the Gumuz (Supplementary Table 4e). *NSUN3* is expressed in the mitochondria as well acting as an RNA methytransferase. It interacts with the anticodon stem loop of mt-tRNA^{Met} and modifies by methylating the cytosine 34. Depletion of *NSUN3* resulted in the alteration of translation of mitochondrial proteins as well as cell growth indicating the

importance of epitranscriptomic modifications for mitochondria protein synthesis [2]. A study reported a patient carrying mutations on *NSUN3* resulting in a non-functional protein. The patient presented mitochondrial disease symptoms with an oxidative phosphorylation deficiency in skeletal muscle [3]. Dihydrofolate Reductase 1 (*DHFRL1*) was previously thought to be a pseudogene but a recent study demonstrated its functionality and expression in humans [4]. This gene has a high homology with *DHFR*, the main gene in charge of maintaining active folate concentrations by the reduction of dihydrofolate to tetrahydrofolate. A study found that *DHFRL1* is localized in the mitochondria that contributes to the *de novo* mitochondrial thymidylate biosynthesis pathway and is essential for mitochondrial DNA (mtDNA) integrity [5]. In addition, expression of *DHFRL1* mRNA was found elevated in Friedrich's ataxia patients that suffer mtDNA damage, indicating that *DHFRL1* could be at the base of limiting mtDNA damage [6].

Among the Somali population we found *CYP3A5* gene among the top unique genes under positive selection (Supplementary Table 4c) *CYP3A5* encodes a member of the cytochrome P450 super family of enzymes. The cytochrome P450 proteins are monooxygenases that catalyse many reactions involved in drug metabolism and synthesis of cholesterol, steroids and other lipids. The encoded protein metabolizes drugs as well as the steroid hormones testosterone and progesterone. Expression of this gene is widely variable among populations, and a single nucleotide polymorphism that affects transcript splicing has been associated with susceptibility to hypertension. A study showed that polymorphisms in *CYP3A* genes could modify the response to dietary methyl-mercury exposure during early life development [7]. Moreover, a case control study in the Han Chinese population showed an association between *CYP3A5* polymorphism and the risk of hypertension [8] but another study with a Ghanaian population found very a small or no association [9]. A study did not find signals of positive selection in *CYP3A5* in the 1000 Genomes populations, thus the signal that we find here might be restricted to this population, making a case of a population-specific adaptation [10].

iHS selection analysis captures recent events of selection – additional examples

All five East African populations have some of the most significant variants in a window containing two long intergenic non-coding RNAs (lincRNAs) *AC105393.1* and *AC105393.2* (Amhara $p<10^{-11}$, Oromo $p<10^{-12}$, Somali $p<10^{-12}$, Wolayta $p<10^{-11}$, Gumuz $p<10^{-14}$). We can see in the 1000 Genomes selection browser [11] that even if the amount of data is low, we also find a signal from CEU, CHB and YRI, indicating a possible event of selection among the human species in general. Both lincRNAs are highly expressed in testis according to the last GTEx release [12].

One of the strongest signals also found in several populations (Amhara, Oromo, Wolayta and Gumuz) falls in the *PPP1R12B* gene. The signal is found in the middle of the gene where we find a high density of exons with variants holding extreme p-values $<10^{-6}$ in all four populations. *PPP1R12B* or *MYPT2* is part of the myosin phosphatase protein complex that is formed by three subunits: a catalytic subunit (PP1c-delta, protein phosphatase 1, catalytic subunit delta), a large regulatory subunit (MYPT, myosin phosphatase target) and small regulatory subunit (sm-M20). There are two isoforms of MYPT, MYPT1 (widely expressed) and MYPT2 (specific to heart, skeletal muscle and brain) [13, 14].

A very interesting signal that we found in the Amhara and Oromo is in a window containing the *TPCN1* gene. *TPCN1* encodes a voltage-gated ion two-pore channel that is activated by NAADP [15]. A recent study found that two-pore channels (TPCs) are crucial for Ebola virus infection by mediating Ebola virus through the endosomal network into the cytoplasm. By inactivating TPC function they prevented Ebola virus to get into the cytoplasm and infect the cell. They further suggested that all filoviruses (e.g. Ebola and Marburg viruses) require TPCs to infect cells [16].

We have also found that Oromo and Wolayta share a window that reaches the 99.99 percentile threshold of significance that contains the gene *KCNH8* (Supplementary Table 5

Figure 5b). It is the highest scoring window encoding a protein in the Oromo population (iHS mean score 3.33 and variants with $p<10^{-7}$) The Amhara and Gumuz do not reach the 99.99 percentile threshold but the 99.9 (reported in Supplementary Table 1). *KCNH8* is mostly expressed in the central nervous system and performs diverse functions including regulation of neurotransmitter release, heart rate, insulin secretion, neuronal excitability, epithelial electrolyte transport, smooth muscle contraction and cell volume [17].

We also report the chromosome 1 histone cluster (*HIST2H*) with significant -log10(p-value) scores among Oromo and Wolayta (mean iHS scores of 2.55 and 3.01 and variants with $p<10^{-3}$ and $p<10^{-4}$ respectively) that is also among the top SFselect candidates in Amhara, Oromo, Somali and Wolayta. It is difficult to understand why a highly conserved gene region could have been under positive selection.

We will now focus on the population-specific protein-coding genes under selection (Supplementary Table 5). For the Gumuz population, we have found three interesting regions containing multiple genes that are good candidates of population specific adaptations. A first region includes *BLOC1S2*, *CHUK* and *PKD2L1*, a second region contains *THEMIS* and a third contains genes encoding T-cell Receptor Alpha Variable (TRAV) locus.

The first of the three regions, spans several windows with mean iHS significant scores (3.74, 3.45, 3.37) and variants with $p<10^{-9}$ and $p<10^{-8}$. This region contains the biogenesis of lysosome-related organelles complex 1 subunit 2 (*BLOS2* or *BLOC1S2*) which is a protein involved in the biogenesis of melanosomes and other lysosome-related organelles. *BLOC1S2* is one of the eight subunits of the protein complex BLOC-1. Five of the eight subunits of BLOC-1 (dysbindin, Capuccino, pallidin, Muted and Snapin) have already been shown to produce strong pigmentation phenotypes in mice [18]. In humans, it is known that mutations in BLOC-1 (particularly in dysbindin, BLOS3 and pallidin) produce Hermansky-

Pudlak syndrome which is a genetically heterogeneous disorder that causes oculocutaneous albinism, prolonged bleeding and pulmonary fibrosis due to the anomalous vesicle trafficking to lysosomes, melanosomes and platelet dense granules [19]. No specific phenotypes have been described yet for mutations in *BLOC1S2* but given the strong pigmentation in the Gumuz population and the high UV-B radiation in Ethiopia it could be an interesting candidate to study in more detail. In the GTeX database, there is a considerable number of significant eQTL in skin.

We also find in the same region the *CHUK* (also named *IKK1 or IKKA*) gene. This gene is a member of the serine/threonine protein kinase family that regulates NF-kB signalling essential for lymphoid organogenesis and adaptive immunity [20]. NF-kB family is highly important for the inflammatory responses; its deregulation can cause a broad range of pathologies (metabolic diseases, chronic inflammatory diseases, autoimmune disorders and cancer).

The second genomic region that is a candidate of population-specific positive selection in the Gumuz contains the thymus-expressed molecule involved in selection (*THEMIS*) gene. This gene plays a crucial role in the positive selection of developing T-cells. It is only expressed in CD4 ad CD8 thymocytes and at a lower expression in lymph nodes and spleen [21]. It acts early in the T-cell receptor signalling cascade by attenuating mild TCR signals that will increase the affinity threshold for activation and allowing positive selection of T cells with naive phenotypes in response to low-affinity self-antigens [22].

Lastly, we found evidence of adaptive selection in the third candidate region specific in the Gumuz. This region contains the T-cell Receptor Alpha Variable (TRAV) locus, which is in charge of antigen recognition. Although the mean iHS score per window do not reach the significance threshold, there are many variants that do reach significance ($p<10^{-6}$).

Another top-scoring signal found only in Oromo contains genes such as sucrase isomaltase (*SI*) (Supplementary Table 5b). *SI* is mostly expressed in the intestinal brush border [23] and it is essential for the digestion of dietary carbohydrates such as starch, sucrose and isomaltose [24]. Congenital sucrase isomaltase deficiency is a rare hereditary disease that causes chronic diarrhoea due to the reduction or absence of *SI* that causes carbohydrate malabsorption [25].

Unbalanced ancestry regions – additional examples

Interestingly, another region of 1 Mb containing an olfactory cluster of genes in the genomic coordinate chr6:27200000-28500000 shows a high proportion of African ancestry, especially in the Amhara (~85%). No clear signals of old or recent selection are found.

Bibliography

1. Pagani L, Schiffels S, Gurdasani D, Danecek P, Scally A, Chen Y, et al. Tracing the Route of Modern Humans out of Africa by Using 225 Human Genome Sequences from Ethiopians and Egyptians. Am J Hum Genet. 2015;96:986–91.

2. Haag S, Sloan KE, Ranjan N, Warda AS, Kretschmer J, Blessing C, et al. NSUN3 and ABH1 modify the wobble position of mt-tRNA ^{Met} to expand codon recognition in mitochondrial translation. EMBO J. 2016;35:2104–19.

3. Van Haute L, Dietmann S, Kremer L, Hussain S, Pearce SF, Powell CA, et al. Deficient methylation and formylation of mt-tRNAMet wobble cytosine in a patient carrying mutations in NSUN3. Nat Commun. 2016;7:12039.

4. McEntee G, Minguzzi S, O'Brien K, Ben Larbi N, Loscher C, O'Fagain C, et al. The former annotated human pseudogene dihydrofolate reductase-like 1 (DHFRL1) is expressed and functional. Proc Natl Acad Sci. 2011;108:15157–62.

5. Anderson DD, Quintero CM, Stover PJ. Identification of a de novo thymidylate biosynthesis pathway in mammalian mitochondria. Proc Natl Acad Sci. 2011;108:15163–8.

6. Haugen AC, Di Prospero NA, Parker JS, Fannin RD, Chou J, Meyer JN, et al. Altered Gene Expression and DNA Damage in Peripheral Blood Cells from Friedreich's Ataxia Patients: Cellular Model of Pathology. PLoS Genet. 2010;6:e1000812.

7. Llop S, Tran V, Ballester F, Barbone F, Sofianou-Katsoulis A, Sunyer J, et al. CYP3A genes and the association between prenatal methylmercury exposure and neurodevelopment. Environ Int. 2017;105:34–42.

8. Li Z, Chen P, Zhou T, Chen X, Chen L. Association between CYP3A5 genotypes with hypertension in Chinese Han population: A case-control study. Clin Exp Hypertens. 2017;39:235–40.

9. Fisher DL, Plange-Rhule J, Moreton M, Eastwood JB, Kerry SM, Micah F, et al. CYP3A5 as a candidate gene for hypertension: no support from an unselected indigenous West African population. J Hum Hypertens. 2016;30:778–82.

10. Dobon B, Rossell C, Walsh S, Bertranpetit J. Is there adaptation in the human genome for taste perception and phase i biotransformation? BMC Evol Biol. 2019;19:39.

11. Pybus M, Dall'Olio GM, Luisi P, Uzkudun M, Carreño-Torres A, Pavlidis P, et al. 1000 Genomes Selection Browser 1.0: a genome browser dedicated to signatures of natural selection in modern humans. Nucleic Acids Res. 2014;42 Database issue:D903-9.

12. Aguet F, Brown AA, Castel SE, Davis JR, He Y, Jo B, et al. Genetic effects on gene expression across human tissues. Nature. 2017;550:204–13.

13. Fujioka M, Takahashi N, Odai H, Araki S, Ichikawa K, Feng J, et al. A New Isoform of Human Myosin Phosphatase Targeting/Regulatory Subunit (MYPT2): cDNA Cloning, Tissue Expression, and Chromosomal Mapping. Genomics. 1998;49:59–68.

14. Okamoto R, Kato T, Mizoguchi A, Takahashi N, Nakakuki T, Mizutani H, et al. Characterization and function of MYPT2, a target subunit of myosin phosphatase in heart. Cell Signal. 2006;18:1408–16.

15. Calcraft PJ, Ruas M, Pan Z, Cheng X, Arredouani A, Hao X, et al. NAADP mobilizes calcium from acidic organelles through two-pore channels. Nature. 2009;459:596–600.

16. Sakurai Y, Kolokoltsov AA, Chen C-C, Tidwell MW, Bauta WE, Klugbauer N, et al. Ebola virus. Two-pore channels control Ebola virus host cell entry and are drug targets for disease treatment. Science. 2015;347:995–8.

17. Zou A, Lin Z, Humble M, Creech CD, Wagoner PK, Krafte D, et al. Distribution and functional properties of human KCNH8 (Elk1) potassium channels. Am J Physiol Physiol. 2003;285:C1356–66.

18. Lee HH, Nemecek D, Schindler C, Smith WJ, Ghirlando R, Steven AC, et al. Assembly and architecture of biogenesis of lysosome-related organelles complex-1 (BLOC-1). J Biol Chem. 2012;287:5882–90.

19. Li W, Zhang Q, Oiso N, Novak EK, Gautam R, O'Brien EP, et al. Hermansky-Pudlak syndrome type 7 (HPS-7) results from mutant dysbindin, a member of the biogenesis of lysosome-related organelles complex 1 (BLOC-1). Nat Genet. 2003;35:84–9.

20. Polley S, Passos DO, Huang D-B, Mulero MC, Mazumder A, Biswas T, et al. Structural Basis for the Activation of IKK1/α. Cell Rep. 2016;17:1907–14.

21. Allen PM. Themis imposes new law and order on positive selection. Nat Immunol. 2009;10:805–6.

22. Fu G, Casas J, Rigaud S, Rybakin V, Lambolez F, Brzostek J, et al. Themis sets the signal threshold for positive and negative selection in T-cell development. Nature. 2013;504:441–5.

23. Traber PG, Wu GD, Wang W. Novel DNA-binding proteins regulate intestine-specific transcription of the sucrase-isomaltase gene. Mol Cell Biol. 1992;12:3614–27.

24. Van Beers EH, Büller HA, Grand RJ, Einerhand AWC, Dekker J. Intestinal brush border glycohydrolases: Structure, function, and development. Crit Rev Biochem Mol Biol. 1995;30:197–262.

25. Marcadier JL, Boland M, Scott CR, Issa K, Wu Z, McIntyre AD, et al. Congenital sucrase-isomaltase deficiency: Identification of a common Inuit founder mutation. CMAJ. 2015;187:102–7.
